# Modelling lifespan reduction in an exogenous damage model of generic disease

**DOI:** 10.1038/s41598-023-43005-0

**Published:** 2023-09-28

**Authors:** Rebecca Tobin, Glen Pridham, Andrew D. Rutenberg

**Affiliations:** 1 Department of Physics and Atmospheric Science, Dalhousie University, Halifax, NS B3H 4R2 Canada; 2 Data Science, Analytics, and Artificial Intelligence (DSAAI) program, Carlton University, Ottawa, K1S 5B6 Canada

**Keywords:** Diseases, Computational models, Statistical physics, thermodynamics and nonlinear dynamics

## Abstract

We model the effects of disease and other exogenous damage during human aging. Even when the exogenous damage is repaired at the end of acute disease, propagated secondary damage remains. We consider both short-term mortality effects due to (acute) exogenous damage and long-term mortality effects due to propagated damage within the context of a generic network model (GNM) of individual aging that simulates a U.S. population. Across a wide range of disease durations and severities we find that while excess short-term mortality is highest for the oldest individuals, the long-term years of life lost are highest for the youngest individuals. These appear to be universal effects of human disease. We support this conclusion with a phenomenological model coupling damage and mortality. Our results are consistent with previous lifetime mortality studies of atom bomb survivors and post-recovery health studies of COVID-19. We suggest that short-term health impact studies could complement lifetime mortality studies to better characterize the lifetime impacts of disease on both individuals and populations.

## Introduction

The emergence of novel diseases—such as COVID-19, Ebola, SARS, Zika, avian flu, or monkeypox—is a worsening trend^1^. Every new disease raises urgent questions about how they could impact infected individuals and the population at large. Yet observational studies offer answers only in retrospect. How can a priori knowledge inform us *before* new diseases are studied and characterized? One approach is to identify potentially universal effects of disease. This approach may also be useful for existing diseases that are not yet fully characterized.

Rapidly increasing mortality with age of infected individuals is a common feature of many infectious diseases^2–9^. For example, short-term mortality due to COVID-19 rises approximately exponentially with age—more than 30-fold from 55 to 85 years^10,11^. Many infectious diseases also exhibit long-term complications, exemplified by post-acute ‘sequelae’ (PAS)—for example, SARS and MERS^12^, Ebola^13^, Zika^14^, ‘long COVID’^15^, and COVID complications^16^. Surprisingly, we do not know the long-term effects of most PAS, how they depend on age, or how they compare to the impact of short-term mortality. This is because there are very few long-term, large-scale studies of the impact of acute disease; most studies are limited to less than 5 years. One notable exception is the study of lifetime mortality impacts of exposure to the atomic bombs at Hiroshima and Nagasaki^17,18^. While this does not represent the effects of disease, it does represent the long-term effects of acute exogenous damage.

Understanding age-effects of disease is particularly important. For example, assuming that short-term mortality is the only impact of acute diseases implies that immunization of older individuals will typically^19^ save more years of life than immunizing younger individuals^11,20^. However, if post-acute health impacts of disease—including PAS—lead to substantial shortened lifespans then immunizing *young* individuals could save more years of life. Resolving these questions of age-effects for individual diseases is not easily done, since lifetime observational studies require many decades.

A promising a priori approach is to computationally model the age-effects of disease. This first requires a model of normal aging. Encouragingly, aging populations exhibit simple and universal behavior. Average human mortality rates exhibit an exponential increase with age known as Gompertz’ law^21^, which is reminiscent of the increased short-term mortality of disease with age. Individual health can be captured by the frailty index, which measures damage and dysfunction^22^. Before death, individuals accumulate damage approximately exponentially with age^23^, leading to worsening individual health^24^. The random but inexorable accumulation of damage during aging can be modelled at the individual level by a complex network of binary health attributes (healthy or not)^25^, where damage propagates stochastically across static links (edges). Such a “Generic Network model” (GNM) of human aging recovers the population-level behaviour of mortality and health^26–29^.

A GNM model provides a dynamical context for propagating damage due to disease. We can model the onset of disease by treating it as an exogenous event that further damages an individual. As such, we can also consider any exogenous damage—and are not specifically limited to disease. While the generic nature of the health attributes in the GNM precludes a detailed study of specific diseases, its generic nature allows us to identify and characterize potentially universal effects of disease in aging individuals.

We will consider the effects of disease timing (onset age), severity, and duration. We will first consider excess mortality (fatality) rates due to disease. To assess the long-term impact of diseases we also need to consider years of life lost due to damage originating from disease. We can use years of life lost within different time horizons to compare short and long-term impacts of disease. We also develop and explore a simplified phenomenological model of how exogenous damage leads to earlier mortality.

## Generic network model (GNM) of disease and exogenous damage

The GNM represents individual health by an undirected scale-free network^30^. Links, defining network topology, are static. Nodes are dynamic binary health attributes—either damaged or not. A summary measure of individual health is the frailty index (*f*)^22,24^, which is the fraction of damaged nodes. An undirected scale-free network is generated using the Barabási–Albert preferential attachment model^31^, with an average node degree $\langle k \rangle$ and scale-free exponent $\alpha _{GNM}$. Nodes are initially undamaged at age $t=0$, but damage at a rate $\Gamma _+ = \Gamma _0 \exp (\gamma _+ f_i)$, where $f_i$ is the fraction of damaged neighbours for node *i*. Damaged nodes repair at a rate $\Gamma _- = (\Gamma _0/R) \exp (\gamma _- f_i)$, though repair has a negligible effect on population statistics in practice. Individual mortality occurs when the two most connected nodes are both damaged. We use previously determined GNM parameters^26,28^ that approximate sex-combined USA population health and mortality statistics^32^ for ages $t \gtrsim 20$: $\langle k \rangle =4$, $\alpha _{GNM}=2.27$, $\Gamma _0$ = 0.00183, $\gamma _+$ = 7.5, with small repair ($\gamma _- = 6.5$ and $R = 3.0$) and $N=10^4$ nodes. For simplicity and clarity we do not use a false-negative correction^26^ to reduce the range of *f* to $[0,1-q]$—i.e. we use $q=0$ and have $f \in [0, 1]$. Stochastic dynamics are exactly sampled^33^. All plotted data corresponds to at least $10^6$ simulated individuals. Errorbars for averages, unless indicated, are smaller than point sizes. All times are in years.

The GNM models damage from all sources that arises during the aging process, including the propagation or amplification of earlier damage. It then captures mortality effects due to that damage. Since the GNM is parameterized from population health and mortality statistics, it implicitly includes many extrinsic events such as disease or injury—the usual stressors of living. As such we expect that the GNM will allow us to model the effects of an individual disease, which we here consider as additional or perturbative to the normal aging process in order to estimate its effect.

**Figure 1:**
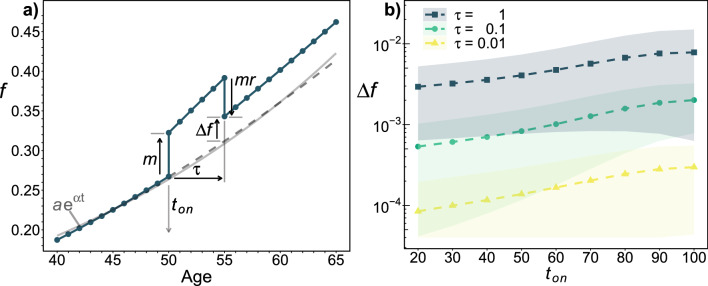
(**a**) Model disease. A disease is represented by exogenous damage of severity *m* inserted at onset time $t_{on}$; a fraction *r* of the original damage is then removed after duration $\tau$. Excess damage that is left at $t_{on}+\tau$ is indicated by $\Delta f$. The average damage vs age, as assessed by the frailty index (*f*, the fraction of damaged nodes within the GNM), for an acute disease with $r=1$, $m=0.05$, $t_{on}=50$ and $\tau =5$ is indicated by the blue points. A control population (with $m=0$) is indicated by the grey dashed line, and is well approximated by an exponential $f=a e^{\alpha t}$ where $a=0.0548\pm 0.0009$, $\alpha = 0.0314\pm 0.0003$, and *t* is the age—as indicated by the solid grey curve. (**b**) Excess damage. Increase in the frailty index at the end of an acute disease, $\Delta f$ at $t=t_{on}+\tau$, with severity $m = 0.02$ vs onset age $t_{on}$, with duration $\tau$ as indicated by legend and $r=1$. The shading indicates the standard deviation of $\Delta f$. All ages and times, in this and other figures, are in years.

We will not model details of the disease process, rather we will simply assume the disease starts (e.g. due to infection) at some onset age $t_{on}$ and lasts for a duration $\tau$. In a similar spirit we will assume that the disease has a fixed severity or magnitude *m*. In terms of the GNM, our model disease damages a fraction *m* of nodes at the onset age $t_{on}$. While formally $m \in [0,1]$, we do not damage already damaged nodes so *m* is kept small. We exclude individuals from analysis who have initial damage $f > 1-m$. For $m \le 0.02$ no individuals are excluded, while for $m = 0.05$ a small fraction ($10^{-4}$) are excluded for $t_{on} \ge 90$. At the end of the disease (at $t_{on}+\tau$) a fraction *r* of the applied damage is removed. The fraction *r* of damage that is removed is a recovery or “resilience” parameter. For acute diseases we typically use $r=1$, while chronic diseases could be modelled with $r=0$ (equivalently, $\tau \rightarrow \infty$). Since we model disease by introducing exogenous damage *m* at time $t_{on}$, and allow for a fraction *r* to be repaired after $\tau$ through resilience, we can use the same model for any exogenous damage. The effect of our model disease is illustrated in Fig. 1a with respect to the frailty index *f*. The control population with no disease is indicated by the grey dashed line. We see that even with $r=1$ there is excess damage $\Delta f$ left in the individual after the end of the disease. This residual damage leads to long-term mortality effects that we characterize. We compare these long-term effects with the short-term acute effects that we also characterize.

We measure long-term mortality using the average reduction in lifespan ($\Delta t_{tot}$) and also by the average years lost within a window of *w* years after the disease ($\Delta t_w$), assuming the mortality rate of the control population after that window. All disease results are with respect to a large control population with no disease ($m=0$). The excess probability of death due to the disease corresponds to an excess infection fatality rate (IFR) as compared to the control population.

**Figure 2:**
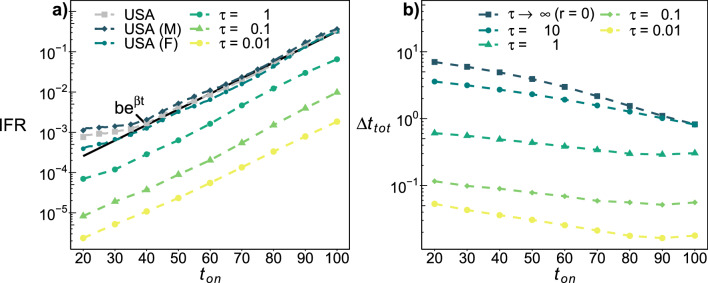
(**a**) Mortality. Excess probability of death during the disease (IFR) vs onset age ($t_{on}$) for acute diseases with duration $\tau$ as indicated, and $m=0.02$. Square grey markers indicates the all-causes mortality rate (per year) vs. age from the U.S. population (2010)^34^. Exponential fit (solid black line): $(4.3 \pm 0.3) \times 10^{-5}\exp {[(0.089\pm 0.001)t_{on}]}$. Male (M) and female (F) sub-populations are as indicated. (**b**) Lifespan reduction. The average total reduction in lifespan due to disease, $\Delta t_{tot}$, vs. onset age $t_{on}$ for severity $m = 0.02$ and duration $\tau$ as indicated by legend, with $r=1$. Chronic disease corresponds to $\tau =\infty$ (or $r=0$).

## GNM results

Our GNM model disease has a significant impact on long-term health, as shown by the average frailty index (*f*) vs age for large simulated populations that received a disease (blue points and solid line) or did not (grey dashed line) in Fig. 1a. With maximal resilience ($r=1$, our default acute disease) all of the damage introduced at $t_{on}$ is removed after $\tau$. Nevertheless excess damage propagates within the GNM and remains at $t_{on}+\tau$, as indicated by $\Delta f$. For a variety of onset ages, and for selected durations $\tau$ as indicated, we show $\Delta f$ in Fig. 1b. We see that $\Delta f$ increases with onset age, and also that the individual variability of propagated damage (indicated by the shaded regions) is large. This reflects the stochastic nature of damage propagation within the GNM.

In Fig. 2a, we show the excess mortality during an acute disease (IFR) vs onset age $t_{on}$. The IFR increases monotonically with $t_{on}$ for all *m* and $\tau$ investigated, and maintains an approximately exponential age dependence similar to the all-causes mortality curve ($\mu$, grey squares). In Fig. 2b, we show the total years lost due to disease ($\Delta t_{tot}$) vs the onset age. Strikingly, we see that the average reduction in lifespan is highest for younger populations (note the log-scale). There are two mechanisms that could contribute to the reduction of lifespan of younger individuals. The first is that mortality during the disease leads to more years of life lost for younger individuals—who have more years left in their life expectancy. The second is that long-term mortality effects could be worse for younger individuals. We can separate these effects by considering different observation windows *w* after the disease.

**Figure 3:**
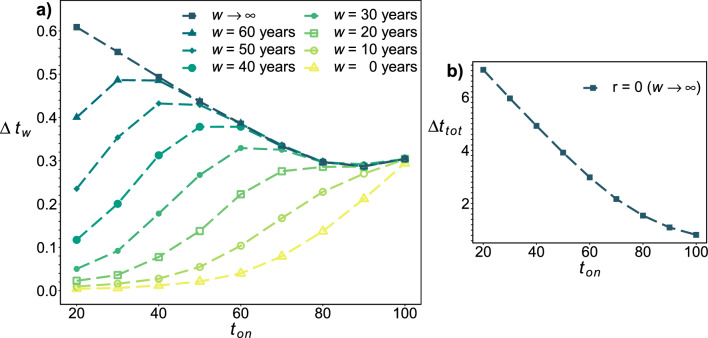
Lifespan reduction for different observation windows. (**a**) The average years lost $\Delta t_w$ vs $t_{on}$ for different observation windows *w* past the end of acute disease (with $r=1$). The effects of mortality during the disease ($w=0$) are largest for older individuals, even though the younger individuals have more lifespan left to lose. The effects of lifetime mortality ($w \rightarrow \infty$) are largest for younger individuals, demonstrating the impact of residual damage. All with $\tau$ = 1 and *m* = 0.02. (**b**) $\Delta t_{tot}$ for a chronic disease ($r=0$). The lifetime effects ($w \rightarrow \infty$) are much larger than in Fig. 3a.

In Fig. 3a, we show the average years lost $\Delta t_w$ within a window of duration *w* after the end of the disease. We account for all excess mortality between $t_{on}$ and $t_{on}+\tau +w$. Just considering deaths during the disease ($w=0$, yellow open triangles), we find that older populations have the largest number of years lost—as observed with, e.g., COVID-19^20^. Even though younger individuals have more lifespan left to lose, it is not enough to offset their much lower IFR. However, for younger ages years lost due to deaths during the disease account for only a small fraction of the total years lost. As we increase *w*, $\Delta t_w$ increases, and its peak shifts towards younger ages. The largest lifetime impact ($\Delta t_\infty \equiv \Delta t_{tot}$, blue squares) is for the youngest individuals, in agreement with Fig. 2b. This effect holds for a wide range of $\tau$ and *m* parameter values, see Supplemental Figs. S2 and S3. Strikingly, the peak (mode) of lifespan impact only moves away from the oldest ages with long observation windows of $w \gtrsim 20$ years. The ratio of lifespan reduction $\Delta t_{tot}/\Delta t_0$ exceeds 100 for the youngest onset ages, and does not strongly depend on duration $\tau$ or severity *m* (Supplemental Fig. S1). The ratio will further increase for lower resilience ($r<1$) since acute mortality, IFR, and acute life lost, $\Delta t_0$, are unchanged but mortality after the disease is increased due to larger residual damage $\Delta f$. For example, in Fig. 3b with $r=0$ we show that $\Delta t_{tot}$ is more than ten-fold larger than with $r=1$.

## Phenomenological model of disease and exogenous damage

While the GNM allows for stochastic and high-dimensional individual health trajectories, the connection between modelling assumptions and phenomenological behavior is obscured by its complexity. A simpler model would be more interpretable—allowing us to see how and when our modelling assumptions lead to the behavior we see. A simpler model would also be easier to generalize. While other mean-field versions of the GNM exist^26,28^, here we develop a simple model that is directly rooted in the observed aging phenomenology: damage accumulates non-linearly with age and this damage drives mortality. The essential simplification here is that the health-state is described only by the average damage—rather than by the many interconnected nodes of the GNM. This phenomenological model complements our network-based simulations using the GNM, and can be easily modified for different phenomenological assumptions.

We start with the observation that the average damage, or frailty index, increases approximately exponentially^35^ with age $f_0(t)=a e^{\alpha t}$. From the GNM, we have $\alpha \approx 0.031$ (and $a \approx 0.055$, see Fig. 1) which is consistent with observational estimates for adults with $t \gtrsim 20$ ($\alpha \approx 0.035 \pm 0.02$^35^). We assume that exogenous damage, such as from disease or injury, forms part of—and behaves similarly to—the damage exhibited during aging. As such it satisfies the differential equation $df/dt = \alpha f$ and any exogenous damage *m* grows exponentially thereafter. By including resilience, we then have simple expressions for the average damage before, during, and after the disease:

1 $$\begin{aligned} f(t)&= {\left\{ \begin{array}{ll} a e^{\alpha t} \, \, &{} t< t_{on},\\ a e^{\alpha t}+m e^{\alpha (t-t_{on})} \, \, &{}t_{on}< t < t_{on}+\tau , \\ a e^{\alpha t} + \Delta f \, e^{\alpha (t-(t_{on}+\tau ))} \, \, &{} t> t_{on}+\tau , \end{array}\right. } \end{aligned}$$

where

2 $$\begin{aligned} \Delta f = m (e^{\alpha \tau }-r) \end{aligned}$$

is the propagated damage at the end of the acute disease (at $t_{end}=t_{on}+\tau$, and with resilience *r*).

This phenomenological damage model is already considerably simplified compared to the GNM: we have a single deterministic health state variable (*f*) rather than $N=10^{4}$ distinct and stochastic health-nodes. By comparing our expression for the propagated damage $\Delta f$ (Eq. 2) with Fig. 1b, we see that the phenomenological model has a single value of $\Delta f$ that is independent of onset age $t_{on}$ while the GNM has a broad range of $\Delta f$ with an average that increases with $t_{on}$—though by much less than the individual variability.

We also need an explicit mortality model. We use the well-established but phenomenological Gompertz law^36^ of $\mu _0 = b e^{\beta t}$, whereby the mortality rate of adults increases exponentially with age. We estimate $\beta \approx 0.089$ (and $b\approx 4.3 \times 10^{-5}$, see Fig. 2a). We then assume that the increasing mortality rate results *only* from the increasing frailty-index *f*(*t*). To obtain the correct time-dependence for mortality from $f_0 \propto e^{\alpha t}$ we have

3 $$\begin{aligned} \mu = b (f/a)^{\beta /\alpha }. \end{aligned}$$

This expression will hold for both the disease and control populations, since by assumption the mortality is expressed only through the health. With a disease, for $t>t_{end}$ we can express this as

4 $$\begin{aligned}{} & {} \mu (t) = b e^{\beta t} \bigg (1+\frac{\Delta f}{f_{end}}\bigg )^{\beta /\alpha }, \end{aligned}$$

where $f_{end} = f_0(t_{end})=a e^{\alpha (t_{on}+\tau )}$ is the control (non-disease) frailty at the end of the disease. Note that a chronic disease corresponds to a disease with no resilience—i.e. $r=0$. A similar expression for the hazard applies during the disease, with the ratio $\Delta f/f_{end}$ replaced by $m/f_{on}$.

**Figure 4:**
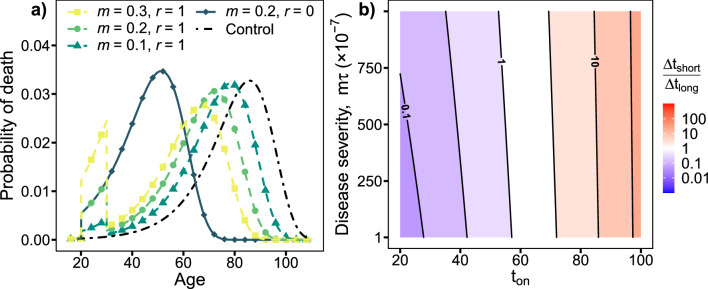
Phenomenological model. (**a**) Effect of varying *m* and *r* on death age. The control distribution (black, dot-dashed line) is shifted towards lower ages by the disease. With resilience (dashed lines), two phases emerge: an acute phase during the disease (ages 20–30) and a chronic phase after the disease ends, due to propagated damage. Each phase contributes to the overall loss of life due to the disease. Without resilience (solid line, $r=0$) the two phases merge into a single short-lived persistent phase. ($\tau = 10$, $t_{on}=20$). (**b**) Acute vs chronic effects. Ratio of expected life lost during acute phase vs chronic phase, $\Delta t_{short}/\Delta t_{long}$. The ratio increases approximately exponentially with increasing age of onset, $t_{on}$, nearly independently of disease severity ($m\tau$) ($\tau = 10^{-3}$, $10^{-4} \le m \le 10^{-1}$, $r=1$).

The lifetime mortality rates, $\mu (t)$, uniquely determine the survival statistics^37^. In Fig. 4a, we present the death age distributions for several disease parameter values. The disease has two lifespan-shortening effects: a short-term, acute effect that increases mortality during the disease, reducing lifespan by $\Delta t_{short}$; and a long-term, chronic effect that shifts the death age distribution to younger ages, further reducing lifespan by $\Delta t_{long}$. In Fig. 4b, we numerically calculate the ratio of acute to chronic effects. As with the GNM, we see that long-term effects dominate for younger individuals whereas short-term effects dominate for older individuals, and are essentially independent of disease severity $m \tau$.

We can also obtain simpler expressions for mortality effects—particularly in the ‘weak’ limit of small *m* and $\tau$. These are useful to develop an understanding of the origins of the effects exhibited by diseases in the GNM.

### Long-term effects

While short-term survival mediates long-term effects, this coupling is small in the weak limit. For simplicity, here we will condition on short-term survival—i.e. assume that individuals are alive at $t_{end}=t_{on}+\tau$ with excess damage $\Delta f$.

Since mortality is determined by health, then the addition of exogenous damage $\Delta f$ at $t_{end}$ effectively ages an individual by $\Delta t_{long}$ where $f_0(t_{end}+\Delta t_{long})=f_0(t_{end})+\Delta f$. This is independent of the form of the mortality law. We obtain

5 $$\begin{aligned}{} & {} \Delta t_{long} = \frac{1}{\alpha } \ln \left( 1+\frac{\Delta f}{f_0(t_{end})}\right) . \end{aligned}$$

This expression neglects a monotonic memory term which is small for young $t_{on}$, but that significantly decreases $\Delta t_{long}$ at old $t_{on}$ (Supplemental Eq. S75). Note that $\Delta t_{long}$ estimates the increase in biological age following disease^35^. Using Eq. (2), and assuming small severities *m* we obtain $\Delta t_{long} \approx \Delta f/(\alpha f_0(t_{end}))$. Further assuming small durations $\tau$ we obtain

6 $$\begin{aligned}{} & {} \Delta t_{long} \approx \frac{m \tau }{f_0(t_{on})} \left( r + \frac{1-r}{\alpha \tau }\right) . \end{aligned}$$

Since mortality only depends on *f*, $\Delta t_{long}$ estimates the long-term reduction in lifespan *after* the survival of mild diseases—excluding any short-term mortality during the disease. Since *f*(*t*) increases with age, $\Delta t_{long}$ is largest in the youngest individuals—independent of disease parameters *m*, $\tau$, and *r*. For imperfect resilience, with $r<1$, chronic effects typically dominate the long-term impact of disease-survivors and $\Delta t_{long} \approx m (1-r)/\left[ \alpha f_0(t_{on}) \right]$; these chronic effects are independent of $\tau$. We observed that COVID-19 has $r < 1$ whereas seasonal flu does not (see below).

### Short-term effects

We can use the hazard $\mu (t)$ in Eq. (4) to solve for the survival probability *S*(*t*), using $dS/dt = - \mu S$ (details are in the supplemental). Conditional on being alive $S=1$ at $t_{on}$ we obtain

7 $$\begin{aligned}{} & {} S(t) = \exp \left[ - \frac{b}{\beta } (f_{on}/a)^{\beta /\alpha }\left( e^{\beta (t-t_{on})}-1\right) \right] , \end{aligned}$$

where $f_{on}$ is the frailty at $t_{on}$. The probability of mortality by the end of an acute disease is $1-S(t_{end})$ therefore we obtain the excess short-term mortality $\Delta p_{death}$ due to the acute disease by the difference in the survival function between using $f_{on}=f_0(t_{on})$ and $f_{on}+m$ at $t_{on}$. For small *m* and $\tau$ we obtain

8 $$\begin{aligned}{} & {} \Delta p_{death} \approx \frac{m\tau \beta }{\alpha }\frac{\mu _0}{f_{on}} = \frac{m\tau \beta }{\alpha }\frac{be^{\beta t_{on}}}{ae^{\alpha t_{on}}}. \end{aligned}$$

We see that $\Delta p_{death} \propto e^{(\beta -\alpha )t_{on}}$ is highest for older individuals since $\beta > \alpha$. This is consistent with the observation of increasing short-term mortality with age in many diseases.

### Comparing short- and long-term effects

To compare short- and long-term effects, we need to estimate the years of life lost due to death during the disease—all within the small *m* and $\tau$ limit. We can approximate the remaining lifespan $\Delta t_D$ from the survival curve by imposing $S(t_{on}+\Delta t_D)=1/e$, this approximates the survival curve as a step function. Using Eq. (7) we obtain $\Delta t_D = \beta ^{-1} \ln (1+\beta /\mu _0(t_{on}))$. The years of life lost during acute disease is then $\Delta t_{short} = \Delta p_{death}\, \Delta t_D$ which gives

9 $$\begin{aligned}{} & {} \Delta t_{short} \approx \frac{m \tau }{f_{on}} \frac{\mu _0}{\alpha } \ln \left( 1+\frac{\beta }{\mu _0}\right) . \end{aligned}$$

In the limit of small *m* and $\tau$, the ratio of short to long-term lifespan effects is then

10 $$\begin{aligned}{} & {} \frac{\Delta t_{short}}{\Delta t_{long}} \approx \frac{\beta }{\alpha } \ln \left( 1+\frac{\beta }{\mu _0}\right) /(\beta /\mu _0), \end{aligned}$$

where we have also allowed for maximal recovery after the disease ($r=1$). Interestingly, this ratio is independent of disease details. We note that $\ln (1+x)/x \approx 1$ for $x \approx 0$ and monotonically decreases towards 0 with increasing $x = \beta /\mu$, i.e. with decreasing age. At large ages $\Delta t_{short}/\Delta t_{long} \approx \beta /\alpha >1$, so that short term mortality during disease affects lifespan more than long-term effects. Conversely, at sufficiently young ages, we expect long-term mortality effects after the disease to have greater impact on lifespan than short-term mortality during the disease. From our estimates of $\alpha$ and $\beta$, $\Delta t_{short}/\Delta t_{long}=1$ for $\mu \approx 0.024$. From all-causes mortality statistics from the U.S. population (Fig. 2a, grey squares) we have $\mu \lesssim 0.024$ for ages $t_{on} \lesssim 70$, implying that $\Delta t_{short} < \Delta t_{long}$ for onset ages $< 70$. So, our phenomenological model indicates that most people would have a greater reduction of lifespan due to premature death long after the disease than from death during the disease. Similar results are observed away from the small *m* and $\tau$ limit (see Fig. 4) and in the GNM (see Fig. 3a).

### Long-term excess relative risk (ERR) and the life-span study (LSS) of atom-bomb survivors

The life-span study (LSS) of approximately 120,000 survivors of the atomic bombs dropped on Nagasaki and Hiroshima has tracked excess lifetime mortality due to radiation exposure for more than 50 years, and found that excess relative risk decreased with age of exposure and was approximately linear with dosage^17,18^. Deaths due to solid-tumor cancer predominate the excess mortality.

Our phenomenological model allows for any source of exogenous damage *m*, not just from disease. We recast it in terms of excess long-term hazard to be able to directly compare with the LSS analysis. Using Eq. (4) with $\tau =0$ we obtain

11 $$\begin{aligned}{} & {} \mu (t) = b e^{\beta t} \left( 1 + \frac{\Delta f}{a} e^{-\alpha t_{on}}\right) ^{\beta /\alpha }. \end{aligned}$$

If we linearize in the hazard in $\Delta f$ we obtain

12 $$\begin{aligned}{} & {} \mu (t) \approx b e^{\beta t}\left( 1 + \frac{\beta }{\alpha }\frac{\Delta f}{a}e^{-\alpha t_{on}} \right) = {\tilde{\mu }}_0(t,\textbf{c}) \left( 1 + \gamma (\textbf{c}) d e^{\theta t_{on}} \right) , \end{aligned}$$

where on the right we show a model of excess relative risk (ERR) from the LSS^17^—here the covariates $\textbf{c}$ such as sex, city, and birth year are indicated ($\text {ERR}\equiv \gamma (\textbf{c}) d e^{\theta t_{on}}$). Qualitatively both the LSS and our approach have excess absolute risk^17^ declining with age of exposure $t_{on}$ and with linear dose-response ($\Delta f$ or *d* in Sv). We can identify $\theta = -\alpha$. Their model estimates $\alpha = 0.045$ (90% CI [0.031, 0.060])^17^, which is consistent with our estimate of 0.031. We suggest that the increased radiation sensitivity at younger exposure ages reported by the LSS^17^ may be a general effect of increased damage sensitivity at younger exposure ages.

Our phenomenological model also suggests different risk models that could be used with LSS data, such as including nonlinear effects with Eq. (11). Using $\alpha =-\theta$ and $\beta =0.089$ (Fig. 2a), we estimate $\Delta f / a = 0.98 d$, where *d* is the exposure dose in Sieverts (Sv)^17^. This implies that the dose and the propagated damage $\Delta f$ are approximately equal, when expressed in natural units. Since survivable doses range up to 5 Sv, the linearized approximation may be worse for younger individuals.

### Parameterizations of COVID-19, influenza and Ebola

Using published IFRs we estimated disease severity, *m*, for COVID-19^38^, influenza^39^ and Ebola^6^, see Table 1. Studies of both COVID-19^40^ and influenza^39^ recorded health in terms pre-disease vs post-recovery frailty, $\Delta f$. This allowed us to estimate the resilience parameter for those diseases, *r*. Each column of Table 1 includes parameter estimates taken from the literature for populations at particular ages, including $\tau$, IFR, and $\Delta f$, together with our phenomenological model estimates for *m* and *r* using Supplemental Eqns. (S2) and (S3), respectively (where possible). Observe that resilience was not significantly different from 1 for influenza, but resilience was significantly lower for COVID-19. This may explain why COVID-19 is observed to have large long-term chronic effects^15,16^: Eq. (6) predicts that $r<1$ effects will dominate the chronic disease effects. See supplemental for details.

Disease severity, *m*, depends on individual robustness—and is used to set the scale for both IFR and $\Delta f$. Note that while $m>1$, we observe physiologically reasonable $\Delta f \ll 1$. We observed that as individuals age, their robustness follows a U-shaped curve: increasing from infancy to adulthood and then decreasing with advanced age (Supplemental Fig. S5). In the case of COVID-19, this decreasing robustness with adult age paralleled the expected changes to frailty, *f*, suggesting a loss of robustness with increasing frailty. Consistent with this, comorbidities both increase the frailty index^22^ and are major risk factors for mortality due to COVID-19^2^.

The frailty index includes both physical and mental deficits^22^. A large UK study found that individuals whom suffered from severe COVID-19 showed reduced cognitive impairment almost $2$ years post-infection comparable to effectively aging $\sim10$ years^41^. Using Eq. (5) we can estimate a generic aging effect from our model. Our $\Delta f$ indicates an effective aging of $\Delta t_{long}=6$ years for a median-aged 57.5 year-old—comparable with the observed cognitive aging^41^.

**Table 1:** Disease parameter estimates for specific ages (95% CI).

	COVID-19	Influenza (hospitalized)	Ebola
Age	65	80.1 (SD 8.7)	16–44
$\tau$ (days)	12	16.8	15.8
IFR	0.017 (0.012–0.027)	0.12 (0.11–0.14)	0.65 (0.64–0.67)
*m*	**1.1** (0.9–1.4)	**2.1** (2.0–2.2)	**5.74** (5.66–5.82)
$\Delta f$	**0.063** (0.046–0.081)	**0.0065** (0.0041–0.0089)	–
*r*	**0.94** (0.93–0.96)	**0.998** (0.997–1.000)	–

Noteworthy values are in [bold].

## Discussion

We have developed and explored a three-parameter model of generic acute disease, which is built upon a generic network model (GNM) of organismal aging (age of onset $t_{on}$, severity *m*, and duration $\tau$). We evaluated short-term mortality outcomes using the excess infection fatality rate (IFR) and long-term mortality outcomes using the average reduction in lifespan due to the disease ($\Delta t_{tot}$). We found that while mortality during acute diseases is highest for older populations, the total reduction in lifespan is highest for younger populations. The majority of the years of life lost for younger populations are due to premature deaths later in life. Older populations have worse short-term outcomes because they have greater frailty *f* (worse health), which leads to a greater likelihood of death during the disease. Younger populations lose more years of life both because there is more to lose and more time for propagated damage $\Delta f$ to impact mortality at the end of life.

Our results are qualitatively consistent with higher short-term mortality for older populations as reported for many acute diseases, including COVID-19^10^, SARS and MERS^2^, influenza^4,5^, Ebola^6^, varicella (chickenpox)^7,9^, and meningococcal disease^8^. While the 1918 (“Spanish”) flu pandemic had much higher than expected mortality for younger adults, this appears to be a special (non-generic) case^42^ partially due to the effects of age-varying immunological history^5,43^.

Long-term impacts due to post-acute sequelae (PAS) are common^12–15,44–49^. We predict that such post-acute effects should increase with acute severity *m*, in qualitative agreement with, e.g., studies of long-COVID^50^. Similar severity dependence is seen in ICU (intensive care unit) survivors^51^. Our disease model is essentially one of exogenous damage, and so should be more general than just acute disease. Long-term studies of hip-fracture survivors have shown significant excess relative risk that is approximately independent of attained age^52,53^ in agreement with our simple phenomenological model (Eq. 11). Atomic bomb survivors provide a unique long-term dataset for exogenous damage due to radiation^17^—with exposure ages ranging from 0 to 60 and with more than 50 years of followup. In agreement with our findings, lifetime risks are greatest for younger exposure ages $t_{on}$.

Aging individuals exhibit changing robustness (resistance to damage) and resilience (recovery from damage)—typically both declining with age^54–56^. Disease frequency typically increases with age^57^, consistent with declining robustness. Robustness and resilience can be considered individual and disease-specific parameters since, e.g., vaccinations or prior exposure increase robustness to infectious disease while, e.g., medical care can improve recovery. Robustness could affect the frequency and/or severity of disease for older individuals (e.g. $t_{on}$ and *m*). Resilience could affect recovery and duration (*r* and $\tau$). Our results are for a fixed severity (*m*) so direct comparisons between ages require caution. Nevertheless, the ratio $\Delta t_{short}/\Delta t_{long}$ is conditioned on the disease occurring, and is largely independent of disease severity (Fig. 4b). The observation that the lifespan impact of disease can be much worse than the acute impact of disease for younger individuals is therefore independent of robustness.

Our model explicitly includes resilience through *r*. Smaller resilience (*r*) should lead to larger $\Delta f$ and thus worse long-term effects. Since resilience is expected to decrease with age^55,56^, we would expect more long-term effects in older individuals. The result would be a smaller ratio of $\Delta t_{short}/\Delta t_{long}$ for older individuals.

Our disease model has no explicit age dependent dynamics, so all effects occur via individual health. We expect that short-term mortality will be worse with either worse health or older ages. Consistent with this, the prognosis of disease generally worsens with a higher frailty index *f*^24,58^. Multiple concurrent diseases are expected to combine additively through *f*, although saturation or exclusion effects may occur for severe or overlapping multimorbidities, respectively. While our phenomenological model has no age effect for $\Delta f$ at a given *m*, our GNM exhibits increasing $\Delta f$ with age. Furthermore, we expect that declining robustness with age (or declining health) will lead to larger *m* and so larger long-term health impacts ($\Delta f$). Such effects are observed. For example, disability following hospitalization increases more with age^59^, and more following ICU admission with frailty^60^. Frailty hinders recovery from influenza^39^. Age is a risk-factor associated with post-COVID-19 conditions^15,50^, and with PAS of chikungunya virus disease^47^.

Consistent with this picture, we observed that our estimates for disease severity, *m*, increased with age. For COVID-19, *m* increased exponentially with age: commensurate with *f* and consistent with a loss of robustness with increasing frailty. Although we did not have data to estimate age-related changes to resilience, we did observe that the seasonal flu showed nearly perfect resilience whereas COVID-19 indicated incomplete recovery ($r<1$). This could help explain the prevalence of COVID-19 PAS^15,16^. Parameterizing additional specific diseases will facilitate future studies to investigate disease-specific effects on lifetime mortality.

Most studies of post-acute mortality effects only have a $w \lesssim 5$ year observation window. We found that $w \gtrsim 20$ year is needed to observe the largest mortality impacts, which we predict occur for smaller onset ages. Larger observation windows *w* are needed. For shorter $w \lesssim 20$ windows, general health measures such as the frailty index *f*^24^ can be used to assess excess damage $\Delta f$ due to the disease. The effective cognitive aging of approximately 10 years due to long COVID-19^41^ is consistent with our generic estimates using Eq. (5). The relative ease with which mental deficits can be measured may make them a convenient way to measure follow up health post-infection.

Our GNM disease model is stochastic and exhibits considerable individual variability in e.g., excess post-acute damage $\Delta f$ (see Fig. 1b). For real diseases, we expect additional variability in the acute severity (*m*). Our models are restricted to adults (with $t \gtrsim 20$), due to similar restrictions on the GNM, frailty *f*, and Gompertz’s law. We expect adult males to experience worse short-term mortality risk, including both acute and chronic effects, due to their higher baseline risk (Supplemental Fig. S6b). This sex-effect is seen in parasite-associated mortality^61^ and most infectious diseases^61,62^.

Our simple phenomenological theory shares with the full disease model our assumptions that residual damage and mortality are determined by health via *f*. Subject to these assumptions, the qualitative agreement of our models indicates the potential universality of our results. From the phenomenological theory we see the key role of the exponential growth rates of mortality and frailty, $\beta$ and $\alpha$ respectively. Empirically we have $\beta >\alpha$, so short-term excess IFR ($\Delta p_{death}$) grows with age. Our phenomenological theory also indicates that post-survivor years of life lost $\Delta t_{long}$ is universally greatest for younger adults—a consequence of $\alpha >0$.

We infer universal aspects of disease through the effects of direct (*m*) and secondary damage ($\Delta f$) in an aging population. We find large long-term effects at young onset ages. Including such age-effects in epidemic models, such as for COVID-19^16,19^, would help us better understand and mitigate the impacts of disease on societies. Researchers typically ask if it is better to vaccinate the old to reduce direct risk, or vaccinate the young to reduce overall infection prevalence^19^. Similarly, cost effectiveness of e.g. rotavirus vaccine^63^ or allocation of COVID-19 vaccine^20^ often only consider mortality during disease. Often neglected are the potential chronic effects due to propagated damage, which we find are worse for the young. Our results could have significant implications for how we prioritize medical interventions across age. Long-term observational studies of health and mortality after acute disease or exposure are needed to better capture lifetime disease impacts.

### Supplementary Information

Supplementary Information.

## Data Availability

The disease model code used to generate the data presented in this paper are available at https://github.com/RebeccaTobin/DiseaseModel. The data used for plots is available on request from A.R.
