# Supplementary material for: Modelling lifespan reduction in an exogenous damage model of generic disease
Source: Sci Rep. 2023 Sep 28;13:16304. doi: 10.1038/s41598-023-43005-0 (PMC10539353; doi:10.1038/s41598-023-43005-0)
Supplement: Supplementary file 1 — Supplementary Information. [file 41598_2023_43005_MOESM1_ESM.pdf]

Supplemental information for “Modelling lifespan reduction in an exogenous damage model of generic disease”

Rebecca Tobin[1,2], **Glen Pridham**[1], **and Andrew D. Rutenberg**[1,*]

[1]Department of Physics and Atmospheric Science, Dalhousie University, Halifax, Nova Scotia, Canada, B3H 4R2
[2]Data Science, Analytics, and Artificial Intelligence (DSAAI) program, Carlton University, Ottawa, Canada, K1S 5B6

S1 Generic network model (GNM) additional results

Fig. S1 shows the ratio of total years of life lost due to the disease, Δt_{tot} divided by years of life lost during the disease, Δt_0, for a variety of onset times t_{on} and disease severities ($m\tau$, with $m = 0.02$). Δt_{tot} includes all effects, whereas Δt_0 includes only the acute effects of the disease. Observe that $\Delta t_{tot}/\Delta t_0$ exceeds 100 for younger individuals.

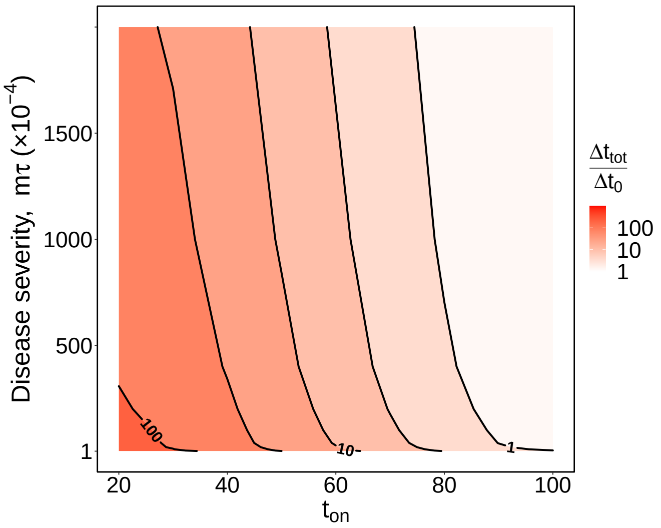

Figure S1. Lifespan reduction ratio for different onset ages and disease severities. Total years of life lost due to disease divided by life lost during only the acute phase. ($m = 0.02$, $10^{-2} \leq \tau \leq 10^2$, $r = 1$)

We performed a sensitivity analysis on the key GNM model parameters: m and τ. The effects of the two parameters were qualitatively similar, and increases to either m or τ smoothly increased both infection fatality rate (IFR), Figure S2, and years of life lost, Figure S3. In the main text we noted the important result that older individuals die more often during disease whereas younger individuals lose more total lifespan. This result holds when tuning either τ or m.

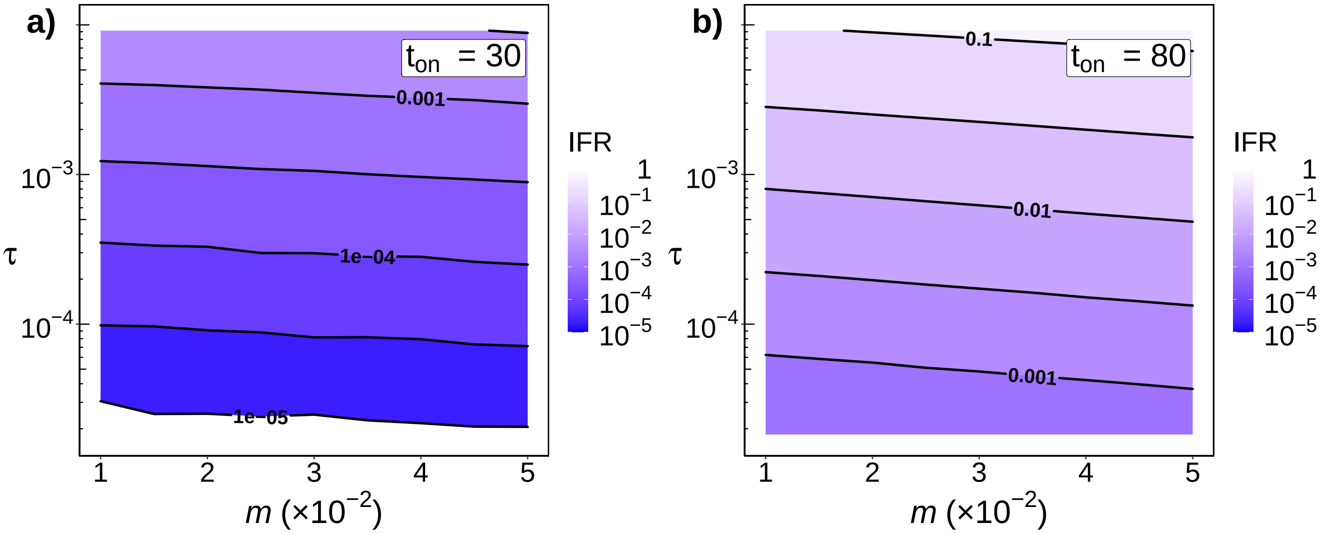

Figure S2. **Infection fatality rate (IFR) as a function of m and τ.** IFR increases smoothly with increasing m or τ. **(a) 30 year old. (b) 80 year old.** Observe the much higher fatality rate for the 80 year old.

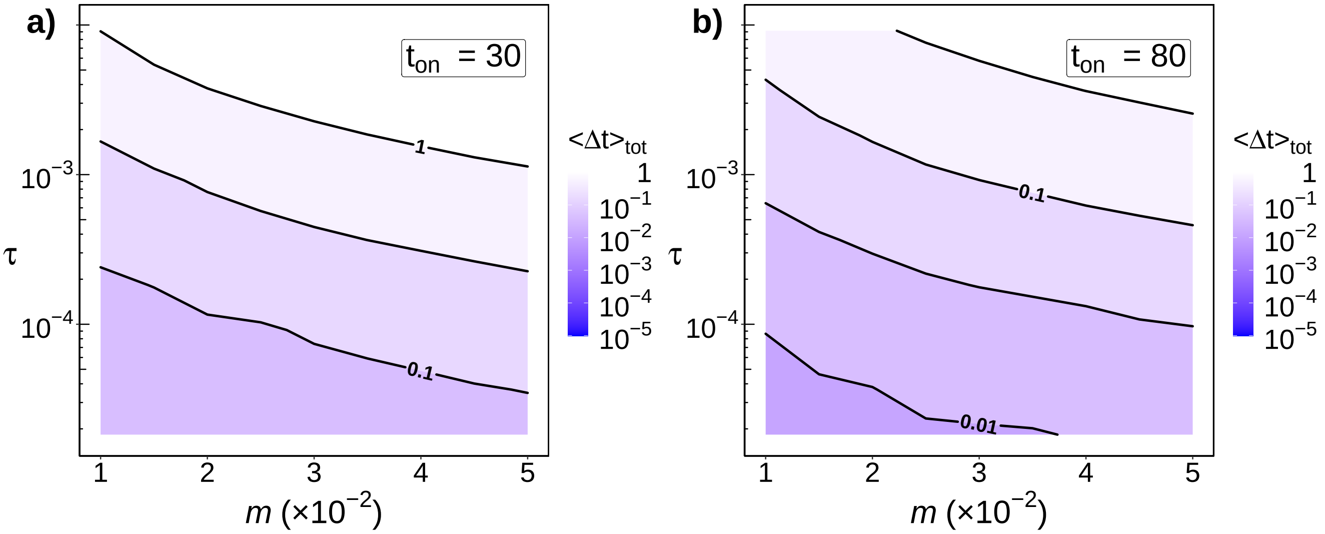

Figure S3. **Total years of life lost as a function of m and τ.** Years of life lost increases smoothly with increasing m or τ. **(a) 30 year old. (b) 80 year old.** Whereas the older individuals died more often during disease, Figure S2, they saw a smaller net loss of life.

S2 Validation for COVID-19, influenza, and ebola

Here we provide details of fitting observed health and mortality data to our phenomenological model, and provide fits for selected diseases. Both the GNM and our phenomenological model are founded on two key hypotheses: (1) acute mortality is due to damage, and (2) this damage should cause secondary propagated damage. Secondary hypotheses are that robustness, via m, and resilience, via r, may vary by disease or due to individual risk factors (including health and age). While the main text primarily explores the key hypotheses, this supplemental section validates the secondary hypotheses using easily available data from influenza, COVID-19, and Ebola. Using acute mortality data we can estimate m (and hence robustness effects). If individual health is followed post-recovery, we can also estimate r (and hence resilience effects).

Several studies have shown evidence of residual or collateral damage post-disease. Increased disability in activities of daily living[1] and increased clinical frailty score[2] have both been observed after COVID-19 recovery. Similar effects are seen in other coronavirus': Middle East Respiratory Syndrome (MERS) and Severe Acute Respiratory Syndrome (SARS). SARS and MERS show long term deficits in fitness capacity and mental health – including increased stress – for up to a year post-recovery[3]. These deficits can lead to collateral, propagated damage due to the negative health effects of stress and dysfunction during the disease together with, e.g., the lack of positive effects of exercise. The main text deals with the consequences of this propagated damage. In general, residual damage may also be due to finite resilience $r < 1$, i.e. acute damage that was not fully recovered from.

Frailty has been observed to increase both after COVID-19[2] and also after hospitalization due to influenza A/B[4]. In Figure S4 we present data from Lees *et al's* study of hospitalizations due to confirmed influenza. They observed a marginally significant increase in the FI (frailty index f) post-influenza. We use this influenza data from Lees *et al*, and data from COVID-19[2], to estimate r (resilience) and m (including any robustness effects) for these diseases. We will also estimate m from Ebola mortality data[5] – where without health information we are unable to estimate r.

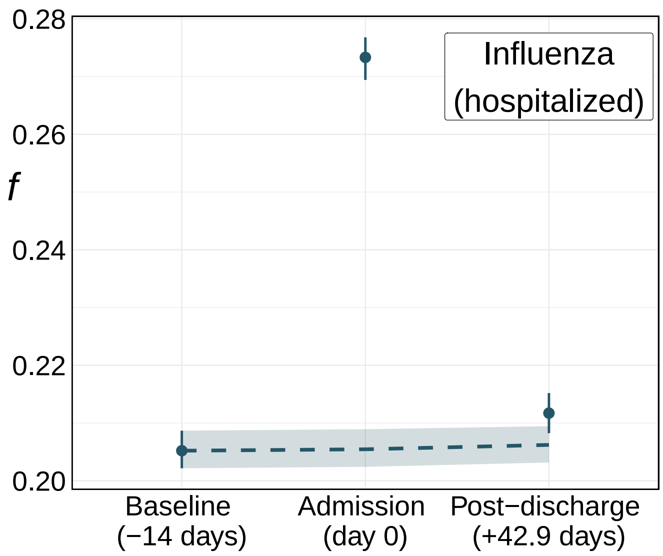

Figure S4. Changes to followup health due to influenza hospitalization. Older individuals (average age 80) were measured for frailty index (FI, f) before, at and after hospitalization for influenza A or B. The band indicates the expected change to FI over the study period for the control group – it is essentially constant. Data were extracted from survivor data in Fig. 2 of Lees *et al*.[4] We approximated that individuals were in the hospital for 12.9 days – this is the average length of stay for COVID-19[6], which is similar to influenza.[7] Error bars are standard error in the mean.

The key statistic for estimating m is the infection fatality rate (IFR). Estimating r requires an additional estimate of Δf. m represents the disease severity and is the fraction of damaged health attributes. Δf represents the residual damage after some fraction, r, of the initial m is repaired — in addition to secondary propagated damage.

The IFR is simply the difference in survival between control and disease groups during the acute period,

$$\begin{aligned} \text{IFR} &\equiv \exp\left[-\int_{t_{on}}^{t_{on}+\tau} \mu(m=0,s)ds\right] - \exp\left[-\int_{t_{on}}^{t_{on}+\tau} \mu(m,s)ds\right] \\ &\equiv S_c(t_{on},t_{on}+\tau) - S_d(t_{on},t_{on}+\tau) \end{aligned} \tag{S1}$$

where the survival of the control, S_c, and disease S_d, are defined by the corresponding terms in the preceding equation. The disease parameters are the age of onset, t_{on}, the disease strength, m, and duration τ. While we do not know the forms of S_c and S_d from the GNM, we can compute analytic forms for the phenomenological model, see Section S3 (S_2 using Eqns. S22 and S23). This model assumes Gompertz' law and also that all mortality occurs due only to changes in the frailty index, f (or "FI").

For the phenomenological model we can algebraically invert Eqn. S1 to yield the m-estimator,

$$
\begin{aligned}
m_{est} &= f_{on}\left(-\frac{\beta}{b}\frac{e^{-\beta(t_{on}+\tau)}}{1-e^{-\beta\tau}}\ln\left[S_c(t_{on},t_{on}+\tau)-\mathrm{IFR}\right]\right)^{\alpha/\beta}-f_{on} \\
&= f_{on}\left(-\frac{\beta}{b}\frac{e^{-\beta(t_{on}+\tau)}}{1-e^{-\beta\tau}}\ln\left[\exp\left(-\frac{b}{\beta}e^{\beta(t_{on}+\tau)}(1-e^{-\beta\tau})\right)-\mathrm{IFR}\right]\right)^{\alpha/\beta}-f_{on},
\end{aligned}
\tag{S2}
$$

where b and β are Gompertz fit parameters from the healthy population, $\alpha \approx 0.031$ is the FI growth exponent and f_{on} is the control-group FI at the start of the disease.

Using published IFR data we estimated m for several diseases using Eqn. S2. m captures both the intrinsic severity of the disease and the individual's resistance to that disease i.e. robustness. Increases to m with age reflect decreases to robustness (and vice versa). In Figure S5 we present m as a function of age for COVID-19 (a) and Ebola (b). As we would expect, robustness increases from infancy to adulthood, causing m to decrease in both diseases. Also expected is that robustness then decreases with increasing age during adulthood, causing m to increase. This increase of m is much faster with COVID-19 (note log-scale) than with Ebola. For COVID-19 the increase of m (decrease of robustness) approximately parallels the increase of the FI (frailty, f). The different behavior of COVID-19 and Ebola supports the hypothesis that robustness is disease dependent. Note that these robustness effects are in addition to the age-effects of acute mortality discussed in the main paper with constant m. The combination of age effects are qualitatively consistent with known mortality risk factors for coronavirus' such as COVID-19, including a strong age dependence and the magnifying effects of comorbidities – which increase f – such as hypertension, diabetes and chronic lung disease[8].

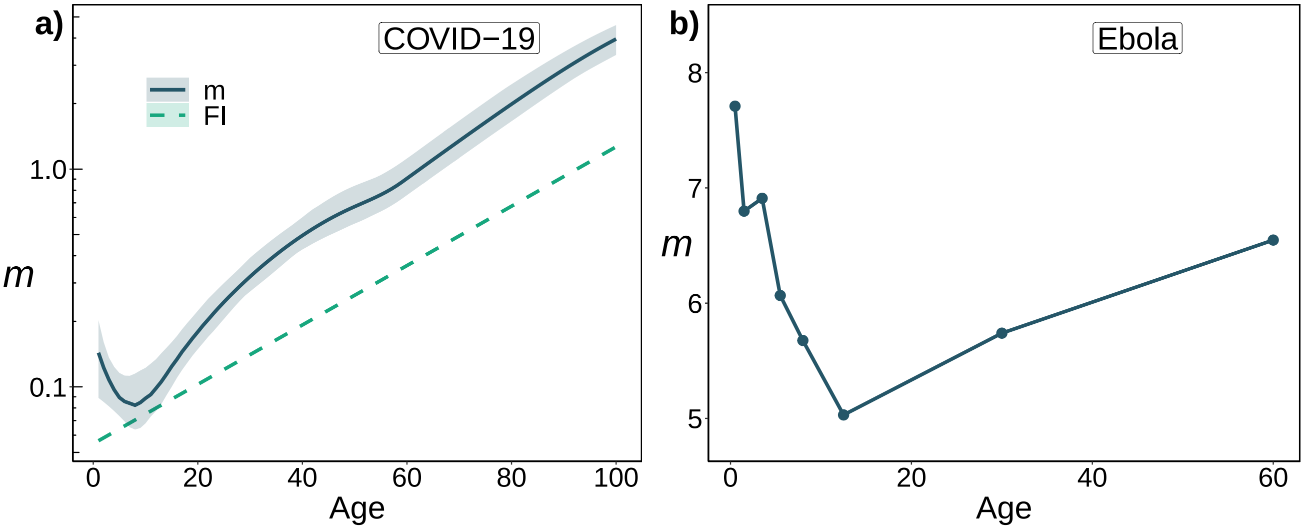

Figure S5. **Disease intensity m estimates, age-dependent.** Using infection fatality rate (IFR) data we estimate m as a function of age, which captures age-related changes to robustness (which decreases as m increases). **(a) COVID-19.** We observed a strong age dependence for COVID-19. For adults, our estimates for m increased exponentially with increasing age (blue solid line), paralleling the expected changes to the FI (green dashed line; same scale). This suggests that robustness may be a function of frailty. Source data: *COVID-19 forecasting team, Lancet, 2022*[9]. **(b) Ebola.** We observed a small increase in susceptibility at young ages in both (note scales) COVID-19 (a) and Ebola (b). For Ebola, the youngest group (< 1 years old) had higher mortality data than the oldest group (45+). Once individuals reach maturity, however, the disease effect, m, seems to be approximately constant with age for Ebola: with at most a linear age-dependence. Finally, note the much stronger effect of Ebola compared to COVID-19, with $m > 1$ at all ages – reflecting the much higher IFR. f captures an individual's health state, so that for very strong diseases with $m > 1$, acutely ill individuals are less healthy than the oldest healthy individual. Source data: *Agua-Agum et al., N Engl J Med, 2015*[5]. The oldest age group (45+) was imputed as age 60 for convenience.

After the disease ends, the residual damage depends on the resilience, r, as

$$\Delta f = m(e^{\alpha\tau} - r), \tag{S3}$$

which permits us to estimate r if both m and Δf are known. Conversely, if we know r then we can estimate Δf.

Parameter estimates for three infectious diseases are reported in Table S1. For influenza and COVID-19 we were able to estimate both the acute effect — via IFR — and the chronic effect via observed changes to patient FI, f. The chronic effect is mediated by the resilience parameter, r, which determines how much endogenous damage persists after the disease. For influenza, we observed that the resilience parameter is very close to 1, suggesting near complete recovery. In contrast, COVID-19 showed far more residual damage with a notably smaller r estimate. This qualitative difference is consistent with the many reported long term side effects for COVID-19, including long COVID[10] and COVID complications[11]. Note that any $r < 1$ can lead to large changes in residual damage Δf – compare Δf_{obs} (observed) and $\Delta f_{r=1}$ (modeled with $r = 1$) in Table S1.

For COVID-19, we estimated m using IFR from the COVID-19 Forecasting Team[9] and compared it to Δf_{obs} from a different study[2]. This latter study measured post-COVID recovery changes to the clinical frailty scale, which was converted to the frailty index f using a linear model trained using data from a third study[12]. In short, non-hospitalized COVID-19 patients saw an increase of 1 in the clinical frailty scale which we estimated as a change of $\Delta f_{obs} = 0.063$. For influenza, we extracted data from Lees *et al.* which captured both mortality and Δf_{obs} for hospitalized influenza A/B patients. The study population were draw from hospitalized patients, which explains why their IFR was so high.

Table S1. Disease Parameter Estimates for Specific Ages (95% CI)

	COVID-19		Influenza (hospitalized)[4]		Ebola[5]	
Age	65		80.1	(SD: 8.7)	16–44$^{(1)}$	
τ (days)	12$^{(2)}$		16.8$^{(3)}$		15.8	
IFR	0.017	(0.012-0.027)	0.12	(0.11-0.14)	0.65	(0.64-0.67)
$m_{est}$$^{(4)}$	1.1	(0.9-1.4)$^{(5)}$	2.1	(2.0-2.2)	5.74	(5.66-5.82)
Δf_{obs}	**0.063**	(0.046-0.081)$^{(6)}$	**0.0065**	(0.0041-0.0089)$^{(7)}$	–	
r_{est}	**0.94**	(0.93-0.96)	**0.998**	(0.997-1.000)	–	
$\Delta f_{r=1}$$^{(8)}$	0.0011	(0.0010-0.0014)	0.0030	(0.0028-0.0032)	0.0078	(0.0077-0.0079)

$^{(1)}$ Estimates are for age 30.
$^{(2)}$ Typical COVID-19 recovery time, 10–14 days[13].
$^{(3)}$ Symptom to hospitalization plus length of time in hospital (mean) for COVID-19[6], which is similar to influenza[7].
$^{(4)}$ m_{est} is the estimated value for m using Eqn. S2.
$^{(5)}$ From fit to acute mortality data[9].
$^{(6)}$ Non-hospitalized COVID-19 patients showed a median 1 point increase in clinical frailty score[2], which was converted to the FI scale using linear regression from a comparison study[12].
$^{(7)}$ Data were extracted from Fig. 2 of Lees *et al.*[4]
$^{(8)}$ $\Delta f_{r=1}$ was computed using m_{est} and Eqn. S3 with $r = 1$.

S3 Phenomenological model

In our generic network model (GNM) the frailty index f is the average number of damaged nodes. Our model of disease increases the frailty index by m at time t_{on} by damaging mN nodes at random (where $N = 10^4$ is the number of nodes). At the end of the disease, a fraction, r, of the damaged nodes are repaired. The model is applied at age t_{on} for duration τ. Mortality occurs when the two most connected nodes of the GNM are simultaneously damaged. Damage promotes damage, thus strongly coupling mortality to frailty. A simple phenomenological model captures this effect and provides analytical expressions for the disease process, which are qualitatively consistent with the GNM.

The essential assumption of the phenomenological model is that the increase in mortality risk with age is solely due to increasing frailty. The mortality risk (hazard) is assumed to obey Gompertz' law,

$$\mu = be^{\beta t} \tag{S4}$$

where b and β are estimated using all-causes mortality data of risk versus age (e.g. Fig. 2a). Frailty represents an individual's state of ill-health, it is equal to the average number of health deficits an individual has. The frailty index is known to increase

exponentially[14] as

$$f = ae^{\alpha t}. \tag{S5}$$

Thus the essential assumption of our model is that

$$\mu = b(e^{\alpha t})^{\beta/\alpha} = b\left(\frac{f}{a}\right)^{\beta/\alpha}. \tag{S6}$$

S3.1 Damage and resilience

Our disease model is to impose exogenous damage. The effect of damaging a fraction of nodes, m, at time t_{on} is to shift the frailty index (FI),

$$\lim_{\varepsilon \to 0^+} f(t_{on} + \varepsilon) = ae^{\alpha t_{on}} + m. \tag{S7}$$

The individual then accumulates damage as

$$\begin{aligned} f(t_{on} < t < t_{on} + \tau) &= (ae^{\alpha t_{on}} + m)e^{\alpha t} \\ &= f(t, m = 0) + m + m(e^{\alpha t} - 1) \\ &= \text{Control FI} + \text{Exogenous FI} + \text{Propagated FI}. \end{aligned} \tag{S8}$$

Where the last two terms were caused by the disease.

Resilience is modelled by repairing a fraction, r of the exogenous damage at the end of the disease. This leaves an effective 'dose' of residual damage,

$$\Delta f = m(e^{\alpha\tau} - r) \tag{S9}$$

that persists after the disease.

The resilience can be generalized to separately recover from either exogenous (r) or propagated (r_{prop}) damage using,

$$\Delta f_{gen}(r, r_{prop}) = m(1 - r) + m(e^{\alpha\tau} - 1)(1 - r_{prop}). \tag{S10}$$

Note that $\Delta f_{gen}(r = r, r_{prop} = 0) = \Delta f$. The generalized resilience modifies Δf and therefore the post-disease mortality (via the hazard rate). The generalized resilience can be used to decouple the acute and chronic phases of the disease by arbitrarily tuning the effects of propagated damage, $m(e^{\alpha\tau} - 1)$, against the effects of direct damage, m. In this supplemental we take $r_{prop} = 0$, but Eqn. S10 can be used in any expression using Δf.

S3.2 Risk formalism

We formalize risk with time-to-event statistics.[15] The death age distribution is defined as the time-to-event probability density function and is simply the product

$$p(t) = \mu(t)S(t), \tag{S11}$$

where $\mu(t)$ is the risk (hazard) defined as the conditional probability of dying between t and $t + dt$ (divided by dt), and $S(t)$ is the probability of surviving to time t.[15] The survival can be calculated using

$$S(t) = \exp\left(-\int_0^t \mu(u)du\right). \tag{S12}$$

Observed that

$$p(t) = -\frac{dS(t)}{dt}, \tag{S13}$$

and conversely,

$$S(t) = 1 - \int_0^t p(u)du. \tag{S14}$$

The conditional distribution given that death occurs after some reference time, t_r, is

$$p(t,m|t>t_r) = \begin{cases} 0 & t \le t_r \\ \frac{p(t,m)}{S(t_r,m)} & t > t_r \end{cases} \tag{S15}$$

where $S(t_r,m)$ ensures normalization, $\int_0^\infty p(t>t_r,m)dt = 1$. The associated survival function is

$$S(t,m|t>t_r) = \begin{cases} 1 & t \le t_r \\ \frac{S(t,m)}{S(t_r,m)} & t > t_r \end{cases} \tag{S16}$$

using $S(t) = 1 - \int_0^t p(t)dt$.

Finally, a useful result for later is to apply integration by parts using $p(t) = -dS/dt$ (Eqn. S13),

$$\begin{aligned}\int_a^b t p(t)dt &= -\int_a^b t\frac{dS}{dt}dt \\ &= aS(a) - bS(b) + \int_a^b S dt.\end{aligned} \tag{S17}$$

Note that

$$\int_0^\infty t p(t)dt = \int_0^\infty S dt. \tag{S18}$$

S3.3 Disease

The effects of the disease on survival can be formalized by calculating the FI, f, as a function of disease parameters. Using Eqn. S6 we can then calculate the mortality risk and therefore the survival using the risk formalism.[15] The disease parameters are

- the severity, m, equal to the increase in FI during the disease,
- the duration, τ,
- the age of onset, t_{on}, and
- the resilience, r, equal to the fraction of m that is repaired at the end of the disease.

For convenience, we define

$$t_{end} \equiv t_{on} + \tau \tag{S19}$$

as the end time of the disease. The control is the special case with $m = 0$.

We can track f because we know how much damage we're adding and therefore we know,

$$f(t) = \begin{cases} ae^{\alpha t} & t < t_{on} \\ (ae^{\alpha t_{on}} + m)e^{\alpha(t-t_{on})} = (f(t_{on}) + m)e^{\alpha(t-t_{on})} = (a + me^{-\alpha t_{on}})e^{\alpha t} & t_{on} \le t < t_{end} \\ ((f(t_{on}) + m)e^{\alpha(t_{end}-t_{on})} - rm)e^{\alpha(t-t_{end})} = f(t,m=0) + \Delta f e^{\alpha(t-t_{end})} = (a + \Delta f e^{-\alpha t_{end}})e^{\alpha t} & t_{end} \le t \end{cases} \tag{S20}$$

where $\Delta f \equiv m(e^{\alpha\tau} - r)$ and $t_{end} \equiv t_{on} + \tau$. Observe that the disease is equivalent to introducing some initial damage, thus increasing the FI by m. Conversely, we can view adding initial damage as aging the individual,

$$f(t) = ae^{\alpha(t+\delta)} \tag{S21}$$

for gained age, δ, defined by Eqn. S21. See Section S3.5 for details.

We can then calculate the hazard using Eqn. S6 to yield,

$$\mu(t) = \begin{cases} b\left(\frac{f(t,m)}{f(t,m=0)}\right)^{\beta/\alpha} e^{\beta t} & t < t_{on} \\ b\left(\frac{(f(t_{on})+m)e^{-\alpha t_{on}}}{a}\right)^{\beta/\alpha} e^{\beta t} = b\left(\frac{f(t,m)}{f(t,m=0)}\right)^{\beta/\alpha} e^{\beta t} & t_{on} \le t < t_{end} \\ b\left(\frac{(f(t_{end},m=0)+\Delta f)e^{-\alpha t_{end}}}{a}\right)^{\beta/\alpha} e^{\beta t} = b\left(\frac{f(t,m)}{f(t,m=0)}\right)^{\beta/\alpha} e^{\beta t} & t_{end} \le t. \end{cases} \tag{S22}$$

where $f(t,m)/f(t,m=0) = f(t,m)\exp(-\alpha t)/a$ is the relative FI of the case vs control. Observe that the ratio $f(t,m)/f(t,m=0)$ has only step-function time-dependence and is piecewise constant: before, during and after the disease.

Using the fact that $f(t,m)/f(t,m=0)$ is piecewise-constant we can easily compute the survival function by breaking up the integral as follows:

$$\begin{aligned} S(t) &\equiv \exp\left(-\int_0^t \mu(u)du\right) \\ &= \exp\left(-\int_0^{\min(t,t_{on})} \mu(u)du\right)\exp\left(-I(t>t_{on})\int_{t_{on}}^{\min(t,t_{end})} \mu(u)du\right)\exp\left(-I(t>t_{end})\int_{t_{end}}^{t} \mu(u)du\right) \\ &= S_1(\min(t,t_{on}))S_2(\min(t,t_{end}))S_3(t) \end{aligned} \tag{S23}$$

where $I(x)$ is the indicator function which is 1 for true and 0 otherwise. Observe that S can be split up into a product of optional terms S_1, S_2 and S_3,

$$S(t) = \begin{cases} S_1(t) & t \le t_{on} \\ S_1(t_{on})S_2(t) & t_{on} \le t \le t_{end} \\ S_1(t_{on})S_2(t_{end})S_3(t) & t_{end} \le t. \end{cases} \tag{S24}$$

Note that $S_1(t)$ is the probability of survival before the disease, $S_2(t)$ is the conditional probability of survival during the disease, and $S_3(t)$ is the conditional probability of survival after the disease. Conditioning on surviving to the start of the disease is easily achieved by setting $S_1(t_{end}) = 1$.

Proof

$$S_1(t \le t_{on}) \equiv \exp\left(-\int_0^t be^{\beta u}du\right) = \exp\left(\frac{b}{\beta}(1-e^{\beta t})\right) = \exp\left(-\frac{\mu(t)}{\beta}(1-e^{-\beta t})\right) \tag{S25}$$

$$S_2(t_{on} < t \le t_{end}) \equiv \exp\left(-\int_{t_{on}}^t b\left(\frac{(f(t_{on})+m)e^{-\alpha t_{on}}}{a}\right)^{\beta/\alpha} e^{\beta u}du\right) = \exp\left(-\frac{\mu(t)}{\beta}(1-e^{-\beta(t-t_{on})})\right) \tag{S26}$$

$$S_3(t \ge t_{end}) \equiv \exp\left(-\int_{t_{end}}^t b\left(\frac{(f(t_{end},m=0)+\Delta f)e^{-\alpha t_{end}}}{a}\right)^{\beta/\alpha} \exp(\beta u)du\right) = \exp\left(-\frac{\mu(t)}{\beta}(1-e^{-\beta(t-t_{end})})\right). \tag{S27}$$

then

$$S(t) = S_1(t) \qquad t \le t_{on} \tag{S28}$$

$$S(t) = S_1(t_{on})S_2(t) \qquad t_{on} \le t \le t_{end} \tag{S29}$$

$$S(t) = S_1(t_{on})S_2(t_{end})S_3(t) \qquad t_{end} \le t. \tag{S30}$$

QED.

Note that the control survival is simply

$$S(t,m=0) = S_1(t). \tag{S31}$$

S3.4 Timescales

Consider the conditional probability of surviving from a reference time t_r to time t,

$$S_r \equiv \exp\left(-\int_{t_r}^t \mu(u)du\right). \tag{S32}$$

It will prove convenient to deal with the characteristic timescale of S_r, that is the time it takes S_r to decay to a threshold value. A characteristic timescale, t_d, is defined by the condition

$$S_n(t_d) = \frac{1}{d}. \tag{S33}$$

For example, $d = 2$ gives the halflife.

For the control case, $m = 0$, the general solution is

$$t_d = \frac{1}{\beta}\ln\left(\frac{\beta}{b}\ln(d) + e^{\beta t_r}\right). \tag{S34}$$

Observe that $t_d > t_r$.

We note the exponential timescale (with $d = e$) is

$$t_e \equiv \frac{1}{\beta}\ln\left(\frac{\beta}{b} + e^{\beta t_r}\right) \tag{S35}$$

for reference time t_r ($t_r = 0$ for S and S_1, $t_r = t_{on}$ for S_2 and $t_r = t_{end}$ for S_3).

We will encounter integrals of the form

$$\int_{t_r}^{\infty} S(u)du. \tag{S36}$$

For $t_r = 0$ this is the average survival time.[15] We will find that the integral is generally a double exponential of the form $\exp(\exp(t))$ and hence is not analytically integrable. We find that a reasonable approximation is

$$\int_{t_r}^{\infty} S(u)du \approx \int_{t_r}^{t_d} 1du = t_d - t_r, \tag{S37}$$

i.e. that the survival function is approximately a step function equal to 1 until some characteristic time, t_d, then 0 afterwards. The approximation is good for survival functions that drop rapidly, which is achieved by a large μ e.g. S_3 for old individuals.

S3.5 Effective Age

Our phenomenological model maps time to damage and vice versa. Hence exogenous damage introduced by the disease has the same effect as aging some period, δ. Hence, we can think of an individual of age t as having *biological age* $t + \delta$, which represents their state of health i.e. they are as healthy as a control individual of age $t + \delta$.

Consider the hazard after the end of the disease,

$$\mu(t > t_{end}) = b\left(\frac{(f(t_{end}, m = 0) + \Delta f)e^{-\alpha t_{end}}}{a}\right)^{\beta/\alpha} e^{\beta t} \tag{S38}$$

using Eqn. S22. Observe that we can write

$$\mu(t > t_{end}) = be^{\beta\delta}e^{\beta t} = be^{\beta(t+\delta)} = \mu(t + \delta, m = 0). \tag{S39}$$

That is, the hazard of the disease is equal to the hazard of the control, shifted by some effective gain in age, δ. Doing the algebra we have

$$\delta = \frac{1}{\alpha}\ln\left(1 + \frac{\Delta f e^{-\alpha t_{end}}}{a}\right) = \frac{1}{\alpha}\ln\left(1 + \frac{\Delta f}{f_{end}}\right) \tag{S40}$$

where f_{end} is the control FI at time t_{end}.

S3.6 Lost lifespan

Exposure to disease will cause a loss of lifespan compared to control. The simplest non-trivial model is to compare the expected loss of lifespan,

$$\begin{aligned}\langle \Delta t \rangle &\equiv \int_0^{\infty} t p(t, m = 0)dt - \int_0^{\infty} t p(t, m)dt \\ &= \int_0^{\infty} S(t, m = 0) - S(t, m)dt\end{aligned} \tag{S41}$$

where the last line comes Eqn. S18, and $\langle \Delta t \rangle$ is the expected number of lost years of life, $p(t, m)$ is the death age distribution, $S(t, m)$ is the survival function, and $m = 0$ denotes the control group. If we consider only individuals whom lived long enough to get the disease then Eqn. S41 is equivalent to the negative of the excess lifetime risk.[16] We leave the expression in its most general form and later set $S_1(t_{on}) = 1$ to condition on surviving to the start of the disease.

We are particularly interested in the acute versus chronic effects of the disease. We define acute/short and chronic/long diseases by splitting the effects of the disease into time intervals $0 \leq t < t_{end}$ (acute/short) and $t \geq t_{end}$ (chronic/long). We derive the expressions in the Sections S3.6.1 and S3.6.2. In short, the key assumptions are (1) the acute/short phase has identical statistics to the disease until $t_{end} = t_{on} + \tau$ and then reverts to the control and (2) conversely, the chronic/long phase has identical statistics to the control until t_{end} then obeys disease statistics. The survival function is causal, $S(t)$ integrates from 0 to t, never past t. The hazard has no memory, it sees only the current f and t. Hence the statistics are also causal since $p(t) = \mu(t)S(t)$. This has implications for normalizing the short and long diseases.

The short (acute) disease distribution is

$$p_{short}(t,m) = \begin{cases} p(t,m) & t < t_{end} \\ p(t,m=0)\frac{S(t_{end},m)}{S(t_{end},m=0)} & t \geq t_{end}. \end{cases} \tag{S42}$$

This ensures that the survival is the same up until the end of the disease for both the acute and full diseases. The ratio of survival functions ensures that $p_{short}(t,m)$ is properly normalized (i.e. $\int_0^\infty p_{short}(t,m)dt = 1$). This can be equivalently written in terms of S_1, S_2 and S_3 as

$$\begin{aligned} S_{1,short}(t,m) &= S_1(t,m=0) = S_1(t,m) \\ S_{2,short}(t,m) &= S_2(t,m) \\ S_{3,short}(t,m) &= S_3(t,m=0), \end{aligned} \tag{S43}$$

where S_1, S_2 and S_3 are defined above (Eqn. S23). That is, the survival curve differs from control only during the acute phase of the disease. See Section S3.6.1 for proof.

Similarly the long distribution is,

$$p_{long}(t,m) = \begin{cases} p(t,m=0) & t < t_{end} \\ p(t,m)\frac{S(t_{end},m=0)}{S(t_{end},m)} & t \geq t_{end}. \end{cases} \tag{S44}$$

This ensures that the survival is the same up until the end of the control for both the chronic and control. The ratio of survival functions ensures that $p_{short}(t,m)$ is properly normalized. This can be equivalently written in terms of S_1, S_2 and S_3 as

$$\begin{aligned} S_{1,long}(t,m) &= S_1(t,m=0) = S_1(t,m) \\ S_{2,long}(t,m) &= S_2(t,m=0) \\ S_{3,long}(t,m) &= S_3(t,m). \end{aligned} \tag{S45}$$

where S_1, S_2 and S_3 are defined above (Eqn. S23). That is, the survival curve differs from control only during the chronic phase of the disease (after the disease). See Section S3.6.2 for proof.

The probability density functions are illustrated in Fig. S6a. The phenomenological model depends on survival parameters from Gompertz law which can be sex-specific – this slightly changes the way males and females experience the disease, Fig. S6b. Our model predicts that males will see higher short-term mortality rates during both the acute and chronic phases, due to their larger baseline mortality. If the disease is severe enough, females will eventually catch up and surpass the males in mortality rate due to their higher Gompertzian slope. Note that males and females also experience disease differently, with males tending to be more prone to infections[17]. This would correspond to different disease-specific parameters for males and females.

The loss of life for short and long effects are then computed using Eqn. S41 and substituting in for $p(t)$. Note that the loss of life has a non-trivial relationship between the full disease, $\langle \Delta t \rangle$ vs the short and long diseases,

$$\begin{aligned} \langle \Delta t \rangle &= \langle \Delta t \rangle_{short} + \langle \Delta t \rangle_{long} \frac{S_2(t_{end},m)}{S_2(t_{end},m=0)} \\ &= \langle \Delta t \rangle_{short} + \langle \Delta t \rangle_{long} - \langle \Delta t \rangle_{long}\left(1 - \frac{S_2(t_{end},m)}{S_2(t_{end},m=0)}\right) \\ &\neq \langle \Delta t \rangle_{short} + \langle \Delta t \rangle_{long} \qquad \text{(overestimates effect)} \end{aligned} \tag{S46}$$

there is an additional corrective term that accounts for the fact that the chronic effects are under-estimated by the full disease due to deaths during the disease. That is, there would be more people to die if the acute portion of the disease never occurred (you can't die during both the acute phase and the chronic phase). Note, however, that if the disease has low lethality then

$$\langle \Delta t \rangle \approx \langle \Delta t \rangle_{short} + \langle \Delta t \rangle_{long} \qquad \text{if } S_2(t_{end},m) \approx S_2(t_{end},m=0). \tag{S47}$$

Which is assured for sufficiently small τ or m.

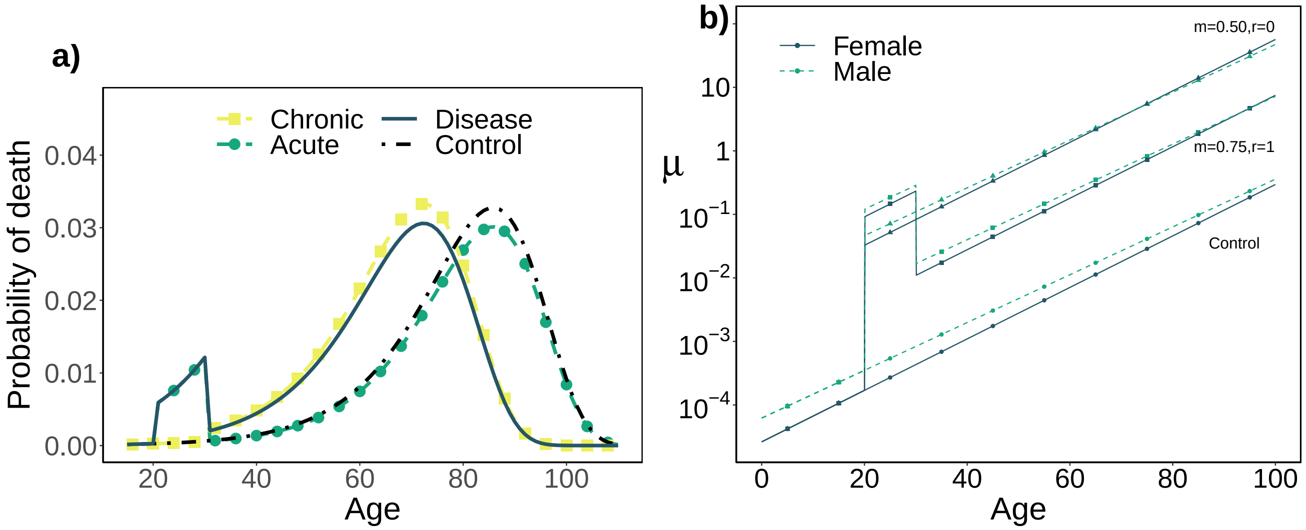

Figure S6. Phenomenological model of disease. a) Acute and chronic phases. Examples for the death age distributions. The disease has two effects (solid line): an acute effect causing a spike of deaths during the disease (ages 20-30) and a chronic effect after the disease that shifts the distribution towards younger ages (ages 30+). The acute phase is defined by having identical statistics to the disease until the end time, t_{end}. Conversely, the chronic phase is defined by having identical statistics to the disease after the end time, t_{end}. Deaths during the disease cause a screening effect, hence neither the chronic and disease nor the acute and control coincide after the disease. ($\tau = 10$, $m = 0.20$, $t_{on} = 20$, $r = 1$). **b) Sex effects of disease on mortality risk, μ.** Males and females are known to have different survival rates, which are captured by our phenomenological model. Males (dashed green lines) have a higher baseline mortality (log-intercept) resulting in worse acute effects whereas females (solid blue lines) have higher slope such that they will eventually catch and exceed males for a sufficiently strong disease. From top to bottom: chronic disease, acute disease and control. The control fits of death probabilities from the Human Mortality Database, 2010 USA life tables[18] are: $\mu_M = (6.0 \pm 0.3) \times 10^{-5} \exp[(0.087 \pm 0.001) \times \text{Age}]$ for males, and $\mu_F = (2.5 \pm 0.2) \times 10^{-5} \exp[(0.094 \pm 0.001) \times \text{Age}]$ for females.

S3.6.1 p_{short} proof

Here we derive Eqn. S42.

By definition, the short (acute) phase has the same statistics as the disease during the disease then reverts to control after the disease. The statistics are governed by the death age distribution hence,

$$p_{short}(t,m) = \begin{cases} Z_1^{-1} p(t, m=0) & t < t_{on} \\ Z_2^{-1} p(t,m) & t_{on} \le t < t_{end} \\ Z_3^{-1} p(t, m=0) & t \ge t_{end}. \end{cases}$$

where we have included normalization constants for complete generality.

By our assumption of identical statistics we should have $Z_1^{-1} p(t, m=0) = p(t < t_{on}, m)$ and $Z_1^{-1} = Z_2^{-1} = 1$, but we also recover these here for completeness. Immediately we have $p(t < t_{on}, m=0) = p(t < t_{on}, m) = p_{short}(t < t_{on}, m)$ as the hazard, μ, is the same prior to the disease and the survival function is causal i.e. it doesn't see the disease coming in the future. Hence $Z_1 = 1$ and $p_{short}(t < t_{on}, m) = p(t < t_{on}, m)$.

Next we consider the survival at t_{end}. The short and complete diseases must have identical survival up until t_{end},

$$\begin{aligned} S(t_{end}, m) &= S_{short}(t_{end}, m), \\ \int_0^{t_{end}} p(t,m)dt &= \int_0^{t_{end}} p_{short}(t,m)dt \\ &= Z_2^{-1} \int_0^{t_{end}} p(t,m)dt \\ \implies Z_2^{-1} &= 1 \end{aligned} \tag{S48}$$

where we have used the relation $S(t) = 1 - \int_0^t p(u)du$ (Eqn. S14).

Now we apply the normalization constraint. For p_{short} to be a valid distribution we must have

$$\begin{aligned}
\int_0^\infty p_{short}(t) = 1 \\
&= \int_0^{t_{end}} p(t,m)dt + \int_{t_{end}}^\infty Z_3^{-1} p(t,m=0)dt \\
&= (1 - S(t_{end},m)) + Z_3^{-1}\left(\int_0^\infty p(t,m=0)dt - \int_0^{t_{end}} p(t,m=0)dt\right) \\
&= (1 - S(t_{end},m)) + Z_3^{-1}\left(1 - (1 - S(t_{end},m=0)\right) \\
\implies (1 - S(t_{end},m)) + Z_3^{-1} S(t_{end},m=0) = 1 \\
\implies Z_3^{-1} = \frac{S(t_{end},m)}{S(t_{end},m=0)}. \qquad \text{(S49)}
\end{aligned}$$

Altogether we have

$$p_{short}(t,m) = \begin{cases} p(t,m) & t < t_{on} \\ p(t,m) & t_{on} \le t < t_{end} \\ p(t,m=0)\frac{S(t_{end},m)}{S(t_{end},m=0)} & t \ge t_{end}. \end{cases}$$

QED.

S3.6.2 p_{long} proof

Here we derive Eqn. S44.

By definition, the long (chronic) phase has the same statistics as the control until the end of the disease state, at which point the statistics switch over to disease. The statistics are governed by the death age distribution hence,

$$p_{long}(t,m) = \begin{cases} Z_1^{-1} p(t,m=0) & t < t_{end} \\ Z_2^{-1} p(t,m) & t \ge t_{end}. \end{cases}$$

where we have included normalization constants for complete generality.

Consider the survival at t_{end}. The long and control must have identical survival up until t_{end},

$$\begin{aligned}
S(t_{end},m=0) &= S_{long}(t_{end},m), \\
\int_0^{t_{end}} p(t,m=0)dt &= \int_0^{t_{end}} p_{long}(t,m)dt \\
&= Z_1^{-1}\int_0^{t_{end}} p(t,m=0)dt \\
\implies Z_1^{-1} &= 1 \qquad \text{(S50)}
\end{aligned}$$

where we have used the relation $S(t) = 1 - \int_0^t p(t)dt$ (Eqn. S14).

Now we apply the normalization constraint. For p_{long} to be a valid distribution we must have

$$\begin{aligned}
\int_0^\infty p_{long}(t) = 1 \\
&= \int_0^{t_{end}} p(t,m=0)dt + \int_{t_{end}}^\infty Z_2^{-1} p(t,m)dt \\
&= (1 - S(t_{end},m=0)) + Z_2^{-1}\left(\int_0^\infty p(t,m)dt - \int_0^{t_{end}} p(t,m)dt\right) \\
&= (1 - S(t_{end},m=0)) + Z_2^{-1}\left(1 - (1 - S(t_{end},m)\right) \\
\implies (1 - S(t_{end},m=0)) + Z_2^{-1} S(t_{end},m) = 1 \\
\implies Z_2^{-1} = \frac{S(t_{end},m=0)}{S(t_{end},m)}. \qquad \text{(S51)}
\end{aligned}$$

Altogether we have

$$p_{long}(t,m) = \begin{cases} p(t,m=0) & t < t_{end} \\ p(t,m)\frac{S(t_{end},m=0)}{S(t_{end},m)} & t \geq t_{end}. \end{cases}$$

QED.

S3.6.3 Δp_{death} Approximation

In this section we obtain a result from the main text, specifically,

$$\Delta p_{death} \equiv \int_{t_{on}}^{t_{end}} \Delta p dt \approx \frac{m\tau\beta\mu_0}{\alpha f_{on}}, \tag{S52}$$

which is the difference between the probability of dying due to the acute phase minus the control. Note that μ_0 is the control group hazard at the start of the disease.

We start by expanding p for small m and τ. For $t_{on} \leq t < t_{end}$,

$$\begin{aligned} p(t,m) &= \mu(t,m)S(t,m) \\ &= b\left(1+\frac{m}{f_{on}}\right)^{\beta/\alpha} e^{\beta t} S_1(t_{on})S_2(t) \\ &\approx be^{\beta t}\left(1+\frac{\beta m}{\alpha f_{on}}\right)S_1(t_{on})(1-\mu(t_{on},m)(t-t_{on})). \end{aligned} \tag{S53}$$

where $1-\mu(t_{on},m)(t-t_{on})$ is the Taylor series of $S_2(t)$ for t near t_{on}.

Now we compute the difference in probability of death during the disease,

$$\begin{aligned} \Delta p_{death} &\equiv \int_{t_{on}}^{t_{end}} p(t,m) - p(t,m=0)dt \\ &\approx \int_{t_{on}}^{t_{end}} be^{\beta t}\left(1+\frac{\beta m}{\alpha f_{on}}\right)S_1(t_{on})(1-\mu(t_{on},m)(t-t_{on})) - be^{\beta t}S_1(t_{on})(1-\mu(t_{on},m=0)(t-t_{on}))dt \\ &= \int_{t_{on}}^{t_{end}} be^{\beta t}\frac{\beta m}{\alpha f_{on}}S_1(t_{on})dt + \mathcal{O}(\tau^2) \\ &\approx S_1(t_{on})be^{\beta t_{on}}\frac{m}{\alpha f_{on}}(e^{\beta\tau}-1) \\ &\approx S_1(t_{on})\frac{m\tau\beta}{\alpha f_{on}}\mu(t_{on},m=0). \end{aligned} \tag{S54}$$

If we condition on individuals living long enough to get the disease then $S_1(t_{on}) = 1$ and we have

$$\Delta p_{death} \approx \frac{m\tau\beta\mu_0}{\alpha f_{on}}. \tag{S55}$$

S3.7 Acute effects

Suppose that the disease group, $m > 0$, experiences the disease but recovers completely after the end of the disease, meaning that they have identical survival statistics after the disease, Eqn. S42. The expected loss of lifespan can then be written as

$$\begin{aligned} \langle \Delta t \rangle_{short} &= \int_0^\infty t(p(t,m=0) - p_{short}(t,m))dt \\ &= \int_0^{t_{on}} t(p(t,m=0) - p_{short}(t,m))dt + \int_{t_{on}}^{t_{end}} t(p(t,m=0) - p_{short}(t,m))dt + \int_{t_{end}}^\infty t(p(t,m=0) - p_{short}(t,m))dt \\ &= \int_0^{t_{on}} t(p(t,m=0) - p(t,m))dt + \int_{t_{on}}^{t_{end}} t(p(t,m=0) - p(t,m))dt + \int_{t_{end}}^\infty t(p(t,m=0) - p(t,m=0)\frac{S(t_{end},m)}{S(t_{end},m=0})dt \end{aligned} \tag{S56}$$

Using integration by parts, this can be re-written in terms of the survival function via Eqn. S17,

$$\begin{aligned}\langle \Delta t \rangle_{short} =& -t_{on} S(t_{on}, m=0) + t_{on} S(t_{on}, m) + \int_0^{t_{on}} S(t, m=0) - S(t,m) dt \\ & + t_{on} S(t_{on}, m=0) - t_{on} S(t_{on}, m) - t_{end} S(t_{end}, m=0) + t_{end} S(t_{end}, m) + \int_{t_{on}}^{t_{end}} S(t, m=0) - S(t,m) dt \\ & + t_{end} S(t_{end}, m=0) - t_{end} S(t_{end}, m=0) \frac{S(t_{end}, m)}{S(t_{end}, m=0}) + \int_{t_{end}}^{\infty} S(t, m=0) - S(t, m=0) \frac{S(t_{end}, m)}{S(t_{end}, m=0)} dt \\ =& \int_0^{t_{on}} S(t, m=0) - S(t,m) dt + \int_{t_{on}}^{t_{end}} S(t, m=0) - S(t,m) dt + \int_{t_{end}}^{\infty} S(t, m=0) - S(t, m=0) \frac{S(t_{end}, m)}{S(t_{end}, m=0)} dt \end{aligned} \tag{S57}$$

It is convenient to work in S_1, S_2 and S_3 since it decouples the disease region from the rest of S (Eqn. S24). Observe

$$\begin{aligned}\langle \Delta t \rangle_{short} =& \int_0^{t_{on}} S_1(t, m=0) - S_1(t,m) dt + S_1 \int_{t_{on}}^{t_{end}} S_2(t, m=0) - S_2(t,m) dt + \\ & + S_1 \int_{t_{end}}^{\infty} S_2(t_{end}, m=0) S_3(t, m=0) - S_2(t_{end}, m) S_3(t, m=0) dt. \end{aligned} \tag{S58}$$

The survival components are identical before, $S_1(m=0) = S_1(m)$, and after, $S_3(m=0) = S_3(m)$, the disease which leads to

$$\langle \Delta t \rangle_{short} = S_1(t_{on}) \int_{t_{on}}^{t_{end}} S_2(t, m=0) - S_2(t,m) dt + S_1(t_{on}) \big(S_2(t_{end}, m=0) - S_2(t_{end}, m) \big) \int_{t_{end}}^{\infty} S_3(t, m=0) dt. \tag{S59}$$

These integrals include double exponential functions which are non-trivial to compute analytically. In the main text we present numerical integration results in Fig. 4b. A perturbative approach reveals useful insights. Suppose the disease occurs for a short period, τ, then we can approximate

$$S_2(t,m) \approx S_2(t_{end}, m) + \left. \frac{dS_2}{dt} \right|_{t_{end}} (t - t_{end}) = S_2(t_{end}, m) - \mu(t_{end}, m) S_2(t_{end}, m)(t - t_{end}) \tag{S60}$$

we then have

$$\begin{aligned}\langle \Delta t \rangle_{short} \approx & S_1(t_{on})(S_2(t_{end}, m=0) - S_2(t_{end}, m))\tau + S_1(\mu(t_{end}, m) S_2(t_{end}, m) - \mu(t_{end}, m=0) S_2(t_{end}, m=0))(-\tau^2/2) \\ & + S_1(t_{on}) \big(S_2(t_{end}, m=0) - S_2(t_{end}, m) \big) \int_{t_{on}}^{\infty} S_3(t, m=0) dt \\ \approx & S_1(t_{on})(S_2(t_{end}, m=0) - S_2(t_{end}, m)) \left(\tau + \int_{t_{end}}^{\infty} S_3(t, m=0) dt \right) \end{aligned} \tag{S61}$$

where we've dropped $O(\tau^2)$ terms due to our assumption that τ is small. Note that $S_3(t_{end}) = 1$ and $S_3(\infty) = 0$. The integral can be approximated as a step function centered at the characteristic time, Eqn. S37, to yield

$$\boxed{\langle \Delta t \rangle_{short} \approx S_1(t_{on}) \Big(S_2(t_{end}, m=0) - S_2(t_{end}, m) \Big) \Big(\tau + (t_e - t_{end}) \Big).} \tag{S62}$$

where t_e is given by Eqn. S35 with $t_r \equiv t_{end}$. Note that $S_1 = 1$ conditions on the population having lived long enough to get the disease, which is standard practice (e.g. excess lifetime risk[16]).

S3.7.1 Weak disease, small m and τ

Starting from Eqn. S62, which assumed small τ, if we further assume m is small then we can write

$$\begin{aligned} S_2(t,m) \approx & S_2(t, m=0) + m \left. \frac{\partial S_2}{\partial m} \right|_{m=0} \\ =& S_2(t, m=0) - \frac{m \mu(t, m=0) S_2(t, m=0)}{\alpha f_{on}} (1 - e^{-\beta(t - t_{on})}) \end{aligned} \tag{S63}$$

where $f_{on} \equiv ae^{\beta t_{on}}$. Thus we can write

$$\langle \Delta t \rangle_{short} \approx S_1(t_{on}) \left(S_2(t_{end}, m=0) - S_2(t_{end}, m=0) + \frac{m\mu(t_{end}, m=0) S_2(t_{end}, m=0)}{\alpha f_{on}} (1 - e^{-\beta(t_{end} - t_{on})}) \right) \left(\tau + (t_e - t_{end}) \right). \tag{S64}$$

Next we apply our earlier condition of small τ to expand the exponential,

$$\langle \Delta t \rangle_{short} \approx S_1(t_{on}) S_2(t_{end}, m=0) \frac{m\beta\tau\mu(t_{end}, m=0)}{\alpha f_{on}} \left(\tau + (t_e - t_{end}) \right). \tag{S65}$$

Substituting in Eqn. S35 we get

$$\begin{aligned} \langle \Delta t \rangle_{short} &\approx S_1(t_{on}) S_2(t_{end}, m=0) \frac{m\beta\tau\mu(t_{end}, m=0)}{\alpha f_{on}} \left(\tau + \left(-t_{end} + \frac{1}{\beta} \ln\left(\frac{\beta}{b} + e^{\beta t_{end}} \right) \right) \right) \\ &= S_1(t_{on}) S_2(t_{end}, m=0) \frac{m\beta\tau\mu(t_{end}, m=0)}{\alpha f_{on}} \left(\tau + \frac{1}{\beta} \ln\left(\frac{\beta}{\mu_{end}} + 1 \right) \right). \end{aligned} \tag{S66}$$

We have already assumed small τ. To lowest order in τ this is

$$\boxed{\langle \Delta t \rangle_{short} \approx S_1(t_{on}) S_2(t_{end}, m=0) \frac{m\tau}{f_{on}} \frac{\mu(t_{end}, m=0)}{\alpha} \ln\left(1 + \frac{\beta}{\mu(t_{end}, m=0)} \right).} \tag{S67}$$

where $S_2(t_{end}, m=0) = 1 - \mu(t_{on}, m=0)\tau \approx 1$ can also be neglected in the strict limit – though we leave it in place (see below). Furthermore, note that $S_1(t_{on}) = 1$ if we condition on individuals being alive at the start of the disease.

S3.8 Chronic effects

Suppose that the disease group, $m > 0$, has identical survival to the control population until time t_{end} when they begin to follow the chronic survival statistics of the disease, Eqn. S44. The expected loss of lifespan can then be written as

$$\begin{aligned} \langle \Delta t \rangle_{long} &= \int_0^{t_{end}} t(p(t, m=0) - p_{long}(t, m))dt + \int_{t_{end}}^{\infty} t(p(t, m=0) - p(t, m))dt \\ &= 0 + \int_{t_{end}}^{\infty} t(p(t, m=0) - p_{long}(t, m=1))dt \end{aligned} \tag{S68}$$

By definition (Eqn S44),

$$\langle \Delta t \rangle_{long} = \int_{t_{end}}^{\infty} t(p(t, m=0) - p(t, m) \frac{S(t_{end}, m=0)}{S(t_{end}, m)})dt \tag{S69}$$

The ratio of survival functions ensures that $p(t,m)$ is properly normalized. Using Eqn. S24 we can write

$$\langle \Delta t \rangle_{long} = S_1(t_{on}, m=0) S_2(t_{end}, m=0) \int_{t_{end}}^{\infty} S_3(t, m=0) - S_3(t, m)dt. \tag{S70}$$

Eqn. S70 can be solved numerically. We seek a more insightful expression. The residual effects of the disease are often weak, motivating us to expand in the effective gain in age, δ (Section S3.5).

We expand S_3 in small gained age, δ, using Eqn. S39,

$$\begin{aligned} S_3(m) &= \exp\left(-\frac{b}{\beta} (1 + \frac{\Delta f}{f_{end}})^{\beta/\alpha} (e^{\beta t} - e^{\beta t_{end}}) \right) \\ &= \exp\left(-\frac{b}{\beta} e^{\beta\delta} (e^{\beta t} - e^{\beta t_{end}}) \right) \\ &\approx S_3(m=0) + \delta \frac{\partial S_3}{\partial \delta}\bigg|_{\delta=0} \\ S_3(m) &\approx S_3(m=0) + \delta \left(\frac{d}{dt} S_3(m=0) + be^{\beta t_{end}} S_3(m=0) \right) \end{aligned} \tag{S71}$$

where we have Taylor expanded near $\delta = 0$. Note that $m = 0$ if and only if $\delta = 0$. Eqn. S70 then simplifies,

$$\begin{aligned}\langle \Delta t \rangle_{long} &\approx S_1(t_{on}, m=0) S_2(t_{end}, m=0) \int_{t_{end}}^{\infty} S_3(t,m=0) - S_3(t,m=0) - \delta\left(\frac{d}{dt} S_3(t,m=0) + b e^{\beta t_{end}} S_3(t,m=0)\right) dt \\ &= \delta S_1(t_{on}, m=0) S_2(t_{end}, m=0) \left(-S_3(t,m=0)\Big|_{ts}^{\infty} - \mu(t_{end}, m=0) \int_{t_{end}}^{\infty} S_3(t,m=0) dt \right) \\ &= \delta S_1(t_{on}, m=0) S_2(t_{end}, m=0) \left(1 - \mu(t_{end}, m=0) \int_{t_{end}}^{\infty} S_3(t,m=0) dt \right). \end{aligned} \tag{S72}$$

We characterize the integral, $\int_{t_{end}}^{\infty} S_3(t,m=0)dt$ in Section S3.8.1, resulting in a pair of constraints,

$$\begin{aligned} 0 < & \mu(t_{end}, m=0) \int_{t_{end}}^{\infty} S_3(t,m=0) dt < 1, \text{ and} \\ 0 < & \mu(t_{end}, m=0) \int_{t_{end}}^{\infty} S_3(t,m=0) dt < \frac{1}{2} \exp\left(\frac{\mu(t_{end}, m=0)}{2\beta}\right) \sqrt{\frac{2\pi \mu(t_{end}, m=0)}{\beta}}. \end{aligned} \tag{S73}$$

At young ages, the upper limit is $\ll 1$, for example at age $t_{end} = 20$ it is 0.07.

In general, $\mu(t_{end}, m=0) \int_{t_{end}}^{\infty} S_3(t,m=0)dt$ does not contribute much at younger ages but becomes increasingly closer to 1 at older ages. A simple way to account for this behaviour is to approximate the integral by a step function (Section S3.4). This assumes the survival function drops very rapidly, which is true for old ages (for young ages the term is small and can be ignored). With this approximation we have,

$$\begin{aligned} \langle \Delta t \rangle_{long} &\approx \delta S_1(t_{on}, m=0) S_2(t_{end}, m=0) \left(1 - \mu(t_{end}, m=0) \int_{t_{end}}^{\infty} I(t_{end} < t_e) dt \right) \\ \langle \Delta t \rangle_{long} &\approx \delta S_1(t_{on}, m=0) S_2(t_{end}, m=0) \Big(1 - \mu(t_{end}, m=0)(t_e - t_{end})\Big) \end{aligned} \tag{S74}$$

where I is an indicator function (1: true, 0: false), and t_e is given by Eqn. S35. Typically we will assume the individual lived long enough to get the disease, $S_1 = 1$. In terms of model parameters we can substitute for δ (Eqn. S40) to get

$$\boxed{\langle \Delta t \rangle_{long} \approx S_1(t_{on}, m=0) S_2(t_{end}, m=0) \frac{1}{\alpha} \ln\left(1 + \frac{\Delta f}{f_{end}}\right) \Big(1 - \mu(t_{end}, m=0)(t_e - t_{end})\Big)}. \tag{S75}$$

Recall f_{end} is the control FI at time $t_{end} = t_{on} + \tau$. The approximation is exact for weak diseases (equivalently: small δ, small m, or small τ with $r = 1$). Note that the second term can never be greater than 1 and is nearly 0 for young t_{end}.

S3.8.1 Characterizing $\int_{t_{end}}^{\infty} S_3(t,m=0)dt$

The integral, $\int_{t_{end}}^{\infty} S_3(t,m=0)dt$, can be characterized by upper and lower bounds. For lower bound, $S_3(t) > 0$ hence the integral must positive. For upper bound, we can expand the inner exponential as a polynomial. Using the definition of S_3, Eqn. S23, and Gompertz law for the hazard we have,

$$\int_{t_{end}}^{\infty} \exp\left(\frac{-b}{\beta}(e^{\beta t} - e^{\beta t_{end}})\right) dt \lesssim \int_{t_{end}}^{\infty} \exp\left(\frac{-b e^{\beta t_{end}}}{\beta}(1 + \beta(t - t_{end}) + \frac{\beta^2}{2}(t - t_{end})^2 - 1)\right) dt \tag{S76}$$

where we use $\lesssim$ to indicate that the right hand side is always larger and will be approximately equal for large t_{end}. Large t_{end} ensures the integrand drops rapidly and hence $t \approx t_{end}$ dominates the integral. To linear order we have,

$$\begin{aligned} \int_{t_{end}}^{\infty} \exp\left(-b e^{\beta t_{end}}(t - t_{end})\right) dt &= \exp\left(b e^{\beta t_{end}} t_{end}\right) \frac{\exp\left(-b e^{\beta t_{end}} t\right)}{-b e^{\beta t_{end}}}\Bigg|_{t_{end}}^{\infty} \\ &= \frac{1}{\mu(t_{end}, m=0)}, \end{aligned} \tag{S77}$$

noting $\mu(t_{end}, m=0) = b e^{\beta t_{end}}$ from Gompertz' law. This constrains

$$0 < \mu(t_{end}, m=0) \int_{t_{end}}^{\infty} S_3(t,m=0) dt < 1. \tag{S78}$$

If we also consider the quadratic term we can set a different upper limit,

$$\begin{aligned}\int_{t_{end}}^{\infty} \exp\left(-be^{\beta t_{end}}(t-t_{end}+\frac{\beta}{2}(t-t_{end})^2)\right)dt &= \int_0^{\infty} \exp\left(-\frac{\beta}{2}be^{\beta t_{end}}(\frac{2}{\beta}u+u^2)\right)du \\ &= \exp\left(\frac{\mu(t_{end},m=0)}{2\beta}\right)\int_0^{\infty} \exp\left(-\frac{1}{2}\mu(t_{end},m=0)\beta(u+1/\beta)^2\right)du \\ &= \exp\left(\frac{\mu(t_{end},m=0)}{2\beta}\right)\sqrt{\frac{2\pi}{\mu(t_{end},m=0)\beta}}\left(1-\Theta(0;-\frac{1}{\beta},\frac{1}{\sqrt{\mu(t_{end},m=0)\beta}})\right)\end{aligned} \tag{S79}$$

where Θ is the integral of a normal distribution with mean $-1/\beta$ and standard deviation $(\mu(t_{end},m=0)\beta)^{-1/2}$. $\Theta \to 1$ as $\mu(t_{end},m=0) \to \infty$ hence the integral becomes small at older t_{end}. Regardless, we know that the normal distribution is symmetrical about the mean and $\beta > 0$ hence $\Theta(0) > 0.5$ and we must have,

$$0 < \mu(t_{end},m=0)\int_{t_{end}}^{\infty} S_3(t,m=0)dt < \frac{1}{2}\exp\left(\frac{\mu(t_{end},m=0)}{2\beta}\right)\sqrt{\frac{2\pi\mu(t_{end},m=0)}{\beta}} \tag{S80}$$

as an additional constraint. At young ages, the upper limit is < 1, for example at age $t_{end} = 20$ it is 0.07.

S3.8.2 Weak disease, small m and small τ

If the disease is sufficiently weak we can make a further approximation of Eqn. S75. Implicitly, by assuming small δ we've already assumed either small m or simultaneously small τ and $r = 1$. Observe we have,

$$\langle\Delta t\rangle_{long} \approx S_1(t_{on},m=0)S_2(t_{end},m=0)\frac{1}{\alpha}\ln\left(1+\frac{m(e^{\alpha\tau}-r)}{f_{end}}\right)\left(1-\mu(t_{end},m=0)(t_e-t_{end})\right). \tag{S81}$$

Substituting in Eqn. S35 for t_e,

$$\langle\Delta t\rangle_{long} \approx S_1(t_{on},m=0)S_2(t_{end},m=0)\frac{1}{\alpha}\ln\left(1+\frac{m(e^{\alpha\tau}-r)}{f_{end}}\right)\left(1-\frac{\mu(t_{end},m=0)}{\beta}\ln\left(1+\frac{\beta}{\mu(t_{end},m=0)}\right)\right). \tag{S82}$$

For small m we can then expand the logarithm,

$$\langle\Delta t\rangle_{long} \approx S_1(t_{on},m=0)S_2(t_{end},m=0)\frac{1}{\alpha}\left(\frac{m(e^{\alpha\tau}-r)e^{-\alpha\tau}}{ae^{\alpha t_{on}}}\right)\left(1-\frac{\mu(t_{end},m=0)}{\beta}\ln\left(1+\frac{\beta}{\mu(t_{end},m=0)}\right)\right). \tag{S83}$$

If we further assume small τ we see,

$$\boxed{\langle\Delta t\rangle_{long} \approx S_1(t_{on},m=0)S_2(t_{end},m=0)\left(\frac{m\tau}{f_{on}}(r+\frac{1-r}{\alpha\tau})\right)\left(1-\frac{\mu(t_{end},m=0)}{\beta}\ln\left(1+\frac{\beta}{\mu(t_{end},m=0)}\right)\right).} \tag{S84}$$

If we consider only individuals whom survived to get the disease then $S_1(t_{on}) = 1$. We can also approximate $S_2(t_{end}) \approx 1-\mu(t_{on})\tau \approx 1$, although we leave it in anticipation of Section S3.9. The $\mu(t_{end},m=0)$ term is small for young t_{on} and can be neglected but reaches unity for the very old, as discussed above (recall that we used $\int_{t_{end}}^{\infty} S_3(t)dt \approx t_e - t_{end}$).

S3.9 Acute-Chronic ratio

We consider the ratio of acute to chronic disease effects,

$$\begin{aligned}\frac{\langle\Delta t\rangle_{short}}{\langle\Delta t\rangle_{long}} &= \frac{S_1(t_{on},m=0)\int_{t_{on}}^{t_{end}} S_2(t,m=0)-S_2(t,m)dt + S_1(t_{on},m=0)\left(S_2(t_{end},m=0)-S_2(t_{end},m)\right)\int_{t_{end}}^{\infty} S_3(t,m=0)dt}{S_1(t_{on},m=0)S_2(t_{end},m=0)\int_{t_{end}}^{\infty} S_3(t,m=0)-S_3(t,m)dt} \\ &= \frac{\int_{t_{on}}^{t_{end}} S_2(t,m=0)-S_2(t,m)dt}{S_2(t_{end},m=0)\int_{t_{end}}^{\infty} S_3(t,m=0)-S_3(t,m)dt} + \frac{\left(S_2(t_{end},m=0)-S_2(t_{end},m)\right)\int_{t_{end}}^{\infty} S_3(t,m=0)dt}{S_2(t_{end},m=0)\int_{t_{end}}^{\infty} S_3(t,m=0)-S_3(t,m)dt}\end{aligned} \tag{S85}$$

For small τ and m we can use Eqn. S67 and Eqn. S84,

$$\frac{\langle \Delta t \rangle_{short}}{\langle \Delta t \rangle_{long}} \approx \frac{S_1(t_{on}) S_2(t_{end}, m=0) \frac{m\tau}{f_{on}} \frac{\mu(t_{end}, m=0)}{\alpha} \ln\left(1 + \frac{\beta}{\mu(t_{end}, m=0)}\right)}{S_1(t_{on}) S_2(t_{end}, m=0) \left(\frac{m\tau}{f_{on}}\left(r + \frac{1-r}{\alpha\tau}\right)\right)\left(1 - \frac{\mu(t_{end}, m=0)}{\beta} \ln\left(1 + \frac{\beta}{\mu(t_{end}, m=0)}\right)\right)}$$

$$\boxed{\frac{\langle \Delta t \rangle_{short}}{\langle \Delta t \rangle_{long}} \approx \frac{\beta \mu(t_{end}, m=0) \ln\left(1 + \frac{\beta}{\mu(t_{end}, m=0)}\right)}{\alpha\left(r + \frac{1-r}{\alpha\tau}\right)\left(\beta - \mu(t_{end}, m=0)\ln\left(1 + \frac{\beta}{\mu(t_{end}, m=0)}\right)\right)}.} \quad \text{(S86)}$$

As discussed in Section S3.8, the μ term in the denominator is always positive and < 1, increasing from a small correction in young ages to order unity by approximately age 100. Hence dropping this term will give a lower limit that's tight (good) at younger ages but poor at older ages.

References

1. Pizarro-Pennarolli, C. *et al.* Assessment of activities of daily living in patients post COVID-19: a systematic review. *PeerJ* **9**, e11026, DOI: 10.7717/peerj.11026 (2021).
2. Müller, I. *et al.* Frailty assessment for COVID-19 follow-up: a prospective cohort study. *BMJ Open Respir Res* **9**, DOI: 10.1136/bmjresp-2022-001227 (2022).
3. Ahmed, H. *et al.* Long-term clinical outcomes in survivors of severe acute respiratory syndrome and Middle East respiratory syndrome coronavirus outbreaks after hospitalisation or ICU admission: A systematic review and meta-analysis. *J. Rehabil. Medicine* **52**, jrm00063, DOI: 10.2340/16501977-2694 (2020).
4. Lees, C. *et al.* Frailty hinders recovery from influenza and acute respiratory illness in older adults. *J. Infect. Dis.* **222**, 428–437, DOI: 10.1093/infdis/jiaa092 (2020).
5. Agua-Agum, J. *et al.* Ebola virus disease among children in west africa. *N. Engl. J. Med.* **372**, 4, DOI: 10.1056/NEJMc1415318 (2015).
6. Faes, C. *et al.* Time between symptom onset, hospitalisation and recovery or death: Statistical analysis of belgian COVID-19 patients. *Int. J. Environ. Res. Public Heal.* **17**, DOI: 10.3390/ijerph17207560 (2020).
7. Ludwig, M., Jacob, J., Basedow, F., Andersohn, F. & Walker, J. Clinical outcomes and characteristics of patients hospitalized for influenza or COVID-19 in germany. *Int. J. Infect. Dis.* **103**, 316–322, DOI: 10.1016/j.ijid.2020.11.204 (2021).
8. Lu, L. *et al.* A comparison of mortality-related risk factors of COVID-19, SARS, and MERS: A systematic review and meta-analysis. *J. Infect.* **81**, e18–e25, DOI: 10.1016/j.jinf.2020.07.002 (2020).
9. COVID-19 Forecasting Team. Variation in the COVID-19 infection-fatality ratio by age, time, and geography during the pre-vaccine era: a systematic analysis. *Lancet* **399**, 1469–1488, DOI: 10.1016/S0140-6736(21)02867-1 (2022).
10. Thompson, E. J. *et al.* Long COVID burden and risk factors in 10 UK longitudinal studies and electronic health records. *Nat. Commun.* **13**, 3528, DOI: 10.1038/s41467-022-30836-0 (2022).
11. Mulberry, N., Tupper, P., Kirwin, E., McCabe, C. & Colijn, C. Vaccine rollout strategies: The case for vaccinating essential workers early. *PLOS Glob. Public Heal.* **1**, e0000020, DOI: 10.1371/journal.pgph.0000020 (2021).
12. Moreno-Ariño, M., Torrente Jiménez, I., Cartanyà Gutiérrez, A., Oliva Morera, J. C. & Comet, R. Assessing the strengths and weaknesses of the clinical frailty scale through correlation with a frailty index. *Aging Clin. Exp. Res.* **32**, 2225–2232, DOI: 10.1007/s40520-019-01450-w (2020).
13. Medlineplus. Mild to moderate COVID-19 - discharge. https://medlineplus.gov/ency/patientinstructions/000976.htm. Accessed: 2023-7-18.
14. Mitnitski, A. & Rockwood, K. Aging as a process of deficit accumulation: Its utility and origin. *Interdiscip. Top. Gerontol. Geriatr.* **40**, 85–98, DOI: 10.1159/000364933 (2015).
15. Moore, D. F. *Applied Survival Analysis Using R* (Springer, Cham, 2016).
16. Thomas, D. *et al.* Definition and estimation of lifetime detriment from radiation exposures: principles and methods. *Heal. Phys.* **63**, 259–272, DOI: 10.1097/00004032-199209000-00001 (1992).

17. Klein, S. L. & Flanagan, K. L. Sex differences in immune responses. *Nat. Rev. Immunol.* **16**, 626–638, DOI: 10.1038/nri.2016.90 (2016).

18. HMD. Human Mortality Database. Max Planck Institute for Demographic Research (Germany), University of California, Berkeley (USA), and French Institute for Demographic Studies (France). www.mortality.org.
